# Position-Specific Analysis and Prediction for Protein Lysine Acetylation Based on Multiple Features

**DOI:** 10.1371/journal.pone.0049108

**Published:** 2012-11-16

**Authors:** Sheng-Bao Suo, Jian-Ding Qiu, Shao-Ping Shi, Xing-Yu Sun, Shu-Yun Huang, Xiang Chen, Ru-Ping Liang

**Affiliations:** 1 Department of Chemistry, Nanchang University, Nanchang, China; 2 Department of Materials and Chemical Engineering, Pingxiang College, Pingxiang, China; University of Alberta, Canada

## Abstract

Protein lysine acetylation is a type of reversible post-translational modification that plays a vital role in many cellular processes, such as transcriptional regulation, apoptosis and cytokine signaling. To fully decipher the molecular mechanisms of acetylation-related biological processes, an initial but crucial step is the recognition of acetylated substrates and the corresponding acetylation sites. In this study, we developed a position-specific method named PSKAcePred for lysine acetylation prediction based on support vector machines. The residues around the acetylation sites were selected or excluded based on their entropy values. We incorporated features of amino acid composition information, evolutionary similarity and physicochemical properties to predict lysine acetylation sites. The prediction model achieved an accuracy of 79.84% and a Matthews correlation coefficient of 59.72% using the 10-fold cross-validation on balanced positive and negative samples. A feature analysis showed that all features applied in this method contributed to the acetylation process. A position-specific analysis showed that the features derived from the critical neighboring residues contributed profoundly to the acetylation site determination. The detailed analysis in this paper can help us to understand more of the acetylation mechanism and can provide guidance for the related experimental validation.

## Introduction

Proteins are created through a biological process called protein biosynthesis. This process begins with transcription from DNA and the splicing of genes into messenger RNA (mRNA) molecules, which are later translated into polypeptides. At the time of translation, a protein can either be active or inactive, and its subsequent activity is usually regulated by chemical modifications referred to as post-translational modifications (PTMs) [1]. Lysine (K) acetylation is an essentially reversible and highly regulated PTM that has been shown to occur in many protein targets, including core histones, approximately 40 transcription factors and over 30 other proteins [2], [3]. This modification is catalyzed by conserved enzymatic machinery composed of lysine acetyltransferases (KATs, also known as histone acetyltransferases (HATs)), which transfer the acetyl-group of acetyl-CoA to the epsilon-amino group of an internal lysine residue, and lysine deacetylases (KDACs, also known as histone deacetylases (HDACs)) that remove the acetyl-groups [4]. Certain KATs have been shown to also acetylate non-histone transcription-related proteins, and acetylation has been shown to play a critical role in human biology and disease. Promising advances have been made recently in developing drug therapies that target HDACs for certain cancers [5]. Of the known PTMs, lysine acetylation has the capacity to destabilize the chromatin polymer through charge neutralization of the basic lysine residues, potentially harboring structural consequences for higher-order chromatin structures [6], [7], [8]. This scenario is not only crucial in the nucleus but is also important for regulating different cytoplasmic processes, including cytoskeleton dynamics, energy metabolism, endocytosis, autophagy, and even signaling from the plasma membrane [9]. There are many individual reports of lysine acetylation sites on proteins that are involved in diverse biological processes, which suggest that acetylation has broad regulatory functions in addition to the few functions that have actively been studied [10].

The full extent of regulatory roles of protein acetylation is still elusive. Importantly, the identification of acetylation sites will be a foundation for understanding the molecular mechanism of protein acetylation. There are several ways to identify potential acetylation sites, such as mass spectrometry [11], the radioactive chemical method [12], and chromatin immunoprecipitation (ChIP) [13]. However, the conventional experimental identification of lysine acetylation substrates is inefficient; it is laborious and has low throughput [14]. Therefore, the prediction of acetylation sites with computational approaches is desirable and necessary. Moreover, the sites that are predicted by computational models, especially models for performing large-scale predictions, could be of interest with respect to general implications for cell biology and biological experiments.

Various computational approaches have been attempted to predict acetylation proteins based on primary sequences [15], [16], [17], [18], [19], [20]. For example, to fully utilize the available information that is extracted from the original sequence and to overcome the disadvantage of highly unbalanced datasets, Xu et al. [18] proposed a novel predicted method called EnsemblePail, which encodes sequences based on improved position weight matrices (PWMs) and then implements an ensemble of support vector machine (SVM) classifiers trained on the “natural” distribution of the data that were extracted from the original sequence data. Basu et al. [14] performed a hierarchical clustering of core histone lysines based on the sequences surrounding each of these given lysines, in which all 56 histone core sequences were aligned to one another, creating a matrix of pairwise alignment scores; then, a hierarchical tree of the histone sequences was generated. Lee et al. [17] proposed a method called N-Ace to recognize acetylated sites on alanine, glycine, lysine, methionine, serine and threonine. Several important features, such as solvent accessibility and physicochemical properties, were considered when implementing the encoding scheme; then, a two-stage SVM was utilized to learn the computational models. Although those methods achieved great progress in predicting acetylation sites, almost all of them gained high specificity but had low sensitivity, and they lacked detailed analysis and explanation for their results. Some drawbacks of these models in this field should be noted. (i) Some proteins with high homology were not excluded. Many studies barely discarded sequence fragments that had high sequence identity and did not consider the sequence identity of the whole protein sequences. (ii) It is well known that the original sequences contain enormous amounts of information; however, the methods of feature extraction in most of the papers were based on a single technique; thus, it is inevitable that some useful information would be missed. (iii) Almost all of the studies investigated only the amino acid residues that continuously surrounded the central acetylation sites. From a structural biological point of view, KATs docking not always binds with the region that is symmetrically balanced around acetylation sites [21], [22], [23]. Therefore, new methods must be established and used for more effective lysine acetylation site identification.

To fully extract information from the original sequence and to discern the amino acid residues that work on the prediction of acetylation sites as accurately as possible, our paper presents a promising method called PSKAcePred. From the consideration of amino acid composition and position information, evolutionary similarity and physicochemical properties in protein sequences, three methods for feature extraction, namely binary encoding (BE), *K*-nearest neighbors (KNN) scores and average accessible surface area (AASA), were employed to effectively mine the information. Meanwhile, the theory of information gain (IG) [24], which is considered to be a type of tool for analyzing the conservatism of amino acid sequences, was utilized to optimize the positions of the amino acid residues that surround the lysine. We demonstrated that our model outperformed general continuous sequence implementations and achieved both high sensitivity (78.02%) and high specificity (81.66%) in 10-fold cross-validation. Here, we present details on the construction of PSKAcePred, the overall performance assessment, and the intensive benchmark experiments against some existing predictors. A user-friendly web interface is now freely available at: http://bioinfo.ncu.edu.cn/inquiries_PSKAcePred.aspx.

## Methods

### Data Collection and Preprocessing

All lysine acetylation data were extracted from UniProtKB/Swiss-Prot [25] (2012_08, www.uniprot.org), CPLA [26] (2012_08, http://cpla.biocuckoo.org/), PhosphoSitePlus [27] (2012_08, www.phosphosite.org), HPRD [28] (2012_08, http://hprd.org/) and SysPTM [29] (2012_08, http://www.biosino.org/SysPTM/) databases, where UniProtKB/Swiss-Prot is a non-redundant protein database and the acetylation protein were retrieved with the keywords “Acetyllysine” and “Experimental”, CPLA is a an integrated database of protein which specifically concentrate to lysine acetylation, and PhosphoSitePlus, HPRD, SysPTM are the systematic resources for post-translational modifications which contain the data of lysine acetylation. After retaining one protein sequence and removing other identical sequences among those five databases, we finally collected 9829 experimental acetylation protein sequences. It is well known that if the datasets are highly homologous, then the accuracy of prediction can be overestimated. Homology reduction allows us to avoid such a bias. Therefore, we clustered the protein sequences with a threshold of 30% identity using the CD-HIT [30] and 5202 proteins were extracted for further preprocessing. An independent dataset was constructed by randomly selecting 10% of all non-homologous protein entries (5202 protein sequences), which consisted of 520 proteins with 1224 acetylation sites. It was used as a benchmark for evaluating our model as well as for comparing it with other published methods. The remaining 4682 proteins with 9815 acetylation sites were used as the training dataset. Thereafter, acetyllysine fragments were extracted as positive set. We used the same type of residue (lysine), excluding known acetylation sites, as the negative set (non-acetylation sites). Although not all of these non-acetylation sites are necessarily true negatives, it is reasonable to believe that a large majority of them are.

For both the positive and negative sets, we defined a local window with each acetylation or non-acetylation site in the middle and several sequence neighbors on each side; the window was denoted by a sequence fragment *x* = (*s_-m_*…*s*_-2_ *s*_-1_ *s*_0_ *s*_1_ *s*_2_…*s_n_*). Because the structural studies have shown that KAT domains coupled with peptide substrates typically do not exceed 14–20 amino acids in length [21], we first chose maximum number of 10 (*m* = 10, *n* = 10) residues that were upstream and downstream of the acetylation or non-acetylation site in such a way that the whole length of the peptide became 21. Subsequently, the highly similar sequence fragments were removed again from the positive set. For two sequence fragments with more than 30% similarity, only one site was kept while the other was discarded. After that, the non-redundant positive set was composed of 9232 acetylated sequence fragments. The non-redundant negative set and independent set were generated using the same approach as for the positive set. The detailed information for data collection was displayed in Supplementary Table S1.

To perform the cross-validation, all of the non-redundant positive samples were selected to be in the positive training set. The balanced negative training set [17] was randomly extracted from the non-redundant negative samples. However, the negative training set, which was randomly selected, might be not sufficiently response to the characteristics of the overall non-redundant negative samples. Therefore, five negative training sets balanced with the positive sets were obtained by random extraction from the non-redundant negative set. The data of training set and independent set of this paper can be obtained at: http://bioinfo.ncu.edu.cn/inquiries_PSKAcePred.aspx.

### Information Gain (IG)

To obtain new information from the data, we require a good measure for the uncertainty of the given data and the uncertainty between the predicting information and the given data. The information entropy from information theory, which was developed by Shannon [31], provides an effective measure of the uncertainty for a given system. It has been confirmed that the physical entropy used in thermodynamics is more or less closely related to the concept of information used in communication theory [31]. Therefore, information gain (IG), which measures the decrease in information entropy when a given variable is used to group values of another (class) variable, can also be considered to be a measure of the degree of ordering [32], [33].

In fact, for a given protein sequence fragment, the conservative property varies from site to site, and some residues near the central site have little contribution to the identification of the acetyllysine sites [23]. Thus, the current problem is how to choose the specific residues that have a positive influence on the predictive models. In our solution we choose a correlation measure based on the information-theoretical concept of IG, a measure of the uncertainty of a random variable. Here, we define the information entropy *H_c_*(*X*) of each amino acid residue in all sequence fragments as the following:
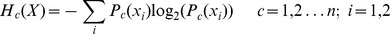
and the entropy of *X* after observing values of another variable *Y* is defined as:



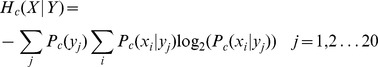
where *n* is the length of the sequence fragments, *P_c_*(*x_i_*) is the prior probabilities for acetylation and non-acetylation sites in all sequence fragments, *P_c_*(*y_j_*) is the probability of the *j*th amino acid occurring in position *c* in those fragments, and *P_c_*(*x_i_*|*y_i_*) is the posterior probabilities of *j*th amino acid in acetylation and non-acetylation sequence fragments. The amount by which the entropy of *X* decreases reflects additional information about *X* provided by *Y* and is called information gain [24].



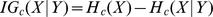



According to the above theory, we can draw the conclusion that the larger the value of IG is, the greater the impact of the corresponding amino acid residue on the acetylation site.

### Amino Acids Binary Encoding (BE) Scheme

The type and position of the amino acid residues are the basic information for a protein sequence. This approach is the simplest and most intuitive algorithm of feature extraction and is based only on the compositional characteristics of the amino acid sequences. To transform protein sequences into numeric vectors, we adopted orthogonal binary vectors. Thus, 20 different amino acids are considered in the binary encoding, which are ordered as ACDEFGHIKLMNPQRSTVWY. Briefly, each amino acid is represented by a 20 dimensional binary vector. For example, amino acid A was expressed as 10000000000000000000, amino acid C as 01000000000000000000, and so on. Therefore, if the length of a protein sequence is *n*, the dimension of the numeric vector is 20*n.

### *K* Nearest Neighbors (KNN) Score

Local sequence clusters often exist around acetylation site because substrate sites of same KATs or KATs family usually share similar patterns in local sequence fragments [23]. To take advantage of such cluster information of local sequence fragments for predicting acetylation sites, we took the local sequence around the acetylation site in a query protein and extracted features from its similar sequences in both positive and negative datasets by a KNN score algorithm [34], [35], [36].

For a query acetylation site, we first find its *K* nearest neighbors in both positive and negative sets according to local sequence similarity. For example, for two local sequence fragments *S_1_* and *S_2_* (the window size is 2*n*+1), define the distance *D*(*S_1_*,*S_2_*) between *S_1_* and *S_2_* as:
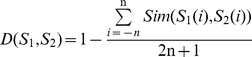


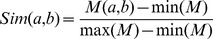
where, *Sim* is derived from the normalized amino acid substitution matrix. *a* and *b* are the two amino acids, *M* is the substitution matrix (BLOSUM62 was used in this paper).

After that, the corresponding KNN score was then extracted as follows: (i) Calculate the average distances from the query sequence fragment *S* to all the training set (contain the positive and negative sets); (ii) Sort the neighbors by the distances and choose the *K* nearest neighbors; (iii) Calculate the percentage of positive neighbors in its *K* nearest neighbors as the KNN score. Last, to take advantage of different properties of neighbors with various similarities, we chose different *K* values to obtain multiple scores. In this paper, *K* was chosen to be 0.025%, 0.05%, 0.1%, 0.2% and 0.4% of the size of the training set, and the five KNN scores were extracted as features for acetylation prediction.

### Amino Acid Physicochemical Property

Physicochemical property is the most intuitive feature for biochemical reactions and is extensively applied in bioinformatics studies [37]. Each of the 20 amino acids has multifaceted properties that are responsible for the specificity and diversity of protein structure and function. A large body of experimental and theoretical research has been performed to characterize different kinds of properties of individual amino acids and to represent them in terms of the numerical index [38], [39]. Version 9.1 of Amino Acid index database (AAindex) [40] has a total of 544 amino acid indices. It includes many published indices that specify the physicochemical properties of amino acids. The amino acid indices with the value “NA” were replaced by 0. Then, the amino acids around the acetylated sites can be encoded according to the values associated with each physicochemical property. All of the 544 physicochemical properties were examined with the default parameters of SVM to determine the prediction ability for model. The properties that were associated with high accuracy were defined as useful features for prediction model. Through testing, the average accessible surface area (AASA) [41] was chosen as the best physicochemical property for acetylation prediction. The cross-validation accuracy of all 544 physicochemical properties is listed in Supplementary Table S2.

### Model Optimization and Evaluation

SVMs were used to evaluate the effects of different types of features. The concept of the SVM is to map the input samples into a higher dimensional space using a kernel function and then to find a hyper-plane that discriminates between the two classes. In this paper, a radial basis function (RBF) was chosen as the kernel function, and two parameters, the penalty parameter *C* and the kernel width parameter *γ*, were tuned based on the training set, using the grid search strategy in LIBSVM. For the actual implementation, we used the LIBSVM package (version 3.1), which can be freely downloaded from http://www.Csie.Ntu.Edu.Tw/~cjlin/libsvm/. To evaluate the predictive performance of the models, cross-validation was performed. In previous studies, the jackknife method was demonstrated to be the most objective validation method [42], [43], but it is time-consuming when the feature-dimension is large. Therefore, 10-fold cross-validation was applied to optimize the parameters, such as the window size and training feature.

The accuracy (Acc), specificity (Sp), sensitivity (Sn) and the Matthews correlation coefficient (MCC) were utilized to assess the predictive performance. In the following formulas, the accuracy denotes the percent of correct prediction in both the positive and negative sets. The sensitivity (the true positive rate) and the specificity (the true negative rate) represent the percentage of the positive set and the negative set that were correctly predicted, respectively. The MCC accounts for the true and false positives and negatives and is usually regarded as a balanced measure that can be used even if the classes are of very different sizes.
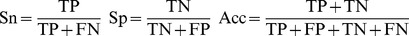


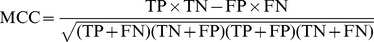



These parameters were defined in terms of the true positive (TP), false negative (FN), true negative (TN), and false positive (FP).

## Results

### Analysis of Different Features

To extract the information comprehensively, we carefully analyzed protein sequence fragments from aspects of amino acid composition and position information, evolutionary similarity and physicochemical properties. The feature encoding scheme included three types of features: amino acids binary encoding (BE), *K* nearest neighbors (KNN), and the average accessible surface area (AASA). Here, we analyzed the distinction between acetylation and non-acetylation from those three features.

#### Analysis of BE features

**Figure 1:**
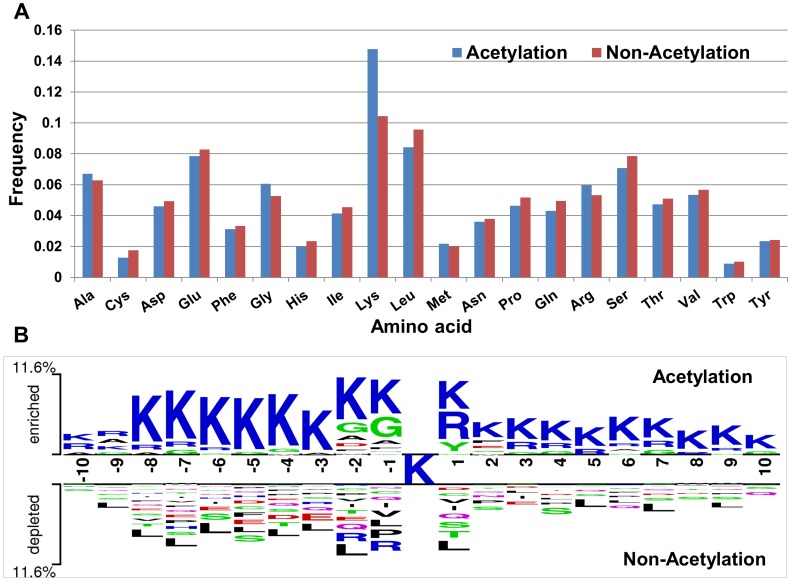
Comparison of sequence information between acetylation sites and non-acetylation sites. (A) Amino acid average composition of acetylation and non-acetylation sites. (B) A two-sample logo of the compositional biases around the acetylation sites compared to the non-acetylation sites.

BE feature reflects the amino acid composition and position information of residues surrounding acetylation sites and non-acetylation sites. In order to analyze specific properties of amino acids, we first calculate the amino acid frequency of both acetylation and non-acetylation samples. As shown in Figure 1A, the amounts of lysine (Lys), arginine (Arg) and Glycine (Gly) that are in acetylation samples are much higher than that of in non-acetylation samples. Indeed, a protein that has a surface that is composed mainly of negatively charged amino acids will bind to a protein with mainly positively charged molecules, such as lysine and arginine [44], [45], [46], [47], [48]. This shows that the acetylation substrates which bind to KATs are much different from non-acetylation. We also adopted a Two Sample Logo [49] (a *P*-value of 0.0001 and a t-test) of 21-mer compositional biases around acetylation conjugation sites compared to non-acetylation conjugation sites. Using this procedure, the amino acid residues that are significantly enriched and depleted around lysine acetylation sites are identified. As shown in Figure 1B, we determine that the characteristics of the residues in the position of −8∼−1 and +1 had significant differences between acetylated and non-acetylated samples. In addition, the ratios of lysine (K) in upstream and arginine (R) in downstream of acetylation sites are higher than those of non-acetylation sites, which is in accordance with the above result. This analysis suggests that sequence profiles of the flanking regions of acetylation sites are more conservative with higher specificity than those of non- acetylation sites.

#### Analysis of KNN features

**Figure 2:**
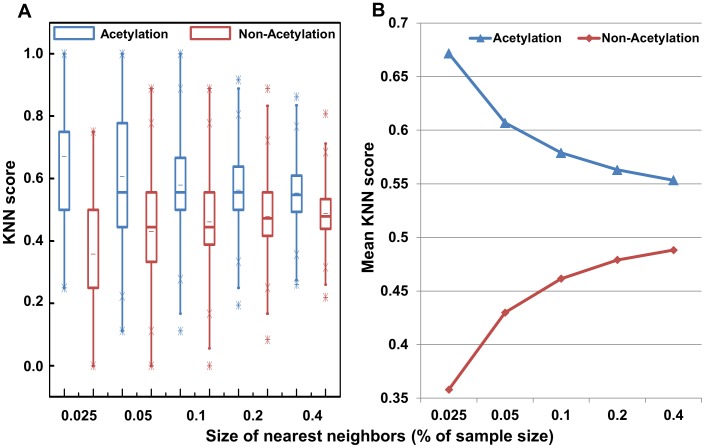
Comparison of KNN scores between acetylation sites and non-acetylation sites. (A) Box plots of KNN scores for acetylation sites and non-acetylation sites. The bottom and top of the box are the 25^th^ and 75^th^ percentiles, respectively. (B) Comparison of mean KNN scores between acetylation sites and non-acetylation sites.

KNN scores measure the evolutionary similarity of the local sequence surrounding a query site between acetylation sequence fragments (positive set) and non-acetylation sequence fragments (negative set). A score greater than 0.5 means the query site is more similar to the acetylation samples; a score smaller than 0.5 means it is more similar to the non-acetylation samples. The larger the KNN score is, the more similar the site is to some known acetylation sites, and thus, the more likely it is an acetylation site. Figure 2 compares the KNN scores of acetylation sites with those of non-acetylation sites. Overall, acetylation sites have larger scores than non-acetylation sites. For acetylation sites, the average KNN scores with different sizes of nearest neighbors are within 0.55∼0.7. Therefore, the local sequences surrounding known acetylation sites are more similar to their nearest neighbors in positive set (excluding self-matches) on average as expected. Note that such similarities are not due to protein homology as the global sequence similarity between any two proteins in our dataset is either insignificant or low [36]. This result confirms that acetylation related clusters exist in acetylation fragments. For non-acetylation sites, the KNN scores are within 0.35∼0.5, which means that the sequences in negative set are more similar to nearest neighbors in negative set. With the increasing of the value of *K*, the gap of KNN scores between acetylation and non-acetylation sites is getting smaller and smaller, which is consistent with the theory of KNN, as shown in Figure 2B. Through testing, when *K* was chosen to be 0.025%, 0.05%, 0.1%, 0.2% and 0.4% of the size of the training set, the predictive result reached its maximum. In short, the KNN scores capture evolutionary similarity information in the local sequence around acetylation sites and hence distinguish them from the background. Therefore, KNN scores are suitable to be used as features for acetylation site prediction.

**Figure 3:**
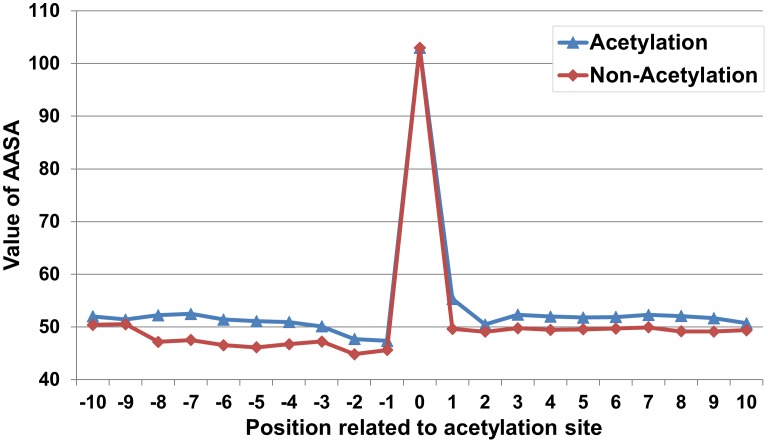
The average accessible surface area (AASA) of residues around acetylation sites and non-acetylation sites.

**Analysis of AASA features:** Pang et al. [50] investigated the structural environment of 8378 incidences of 44 types of PTMs and found a side-chain of amino acid that undergoes post-translational modification (PTM) prefers to be accessible on the surface of a protein. Therefore, the solvent accessibility of amino acid residues surrounding the acetylation sites may be adapted when distinguishes between the acetylation site and non-acetylation sites. In this section, we will demonstrate the effectiveness of AASA as features for acetylation site prediction. Figure 3 plots the AASA formed from the 21-mer acetylation sites in the constructed data set. Most of the acetylation or non-acetylation sites (0 position) are located in the highly ASA, which is consistent with those data reported in the literature [50]. The average AASA of neighborhood residues are 47 to 55 for acetylation sites. The fluctuant range of AASA of residues surrounding acetylation sites is bigger than that of non-acetylation sites. This implies that the acetylation processing might have occurred where the structural surroundings are relatively large variation range. The AASA that surrounds the acetylation sites exceed that around non-acetylation sites, especially in the −8, −7, −6, −5 and −4 positions. Generally speaking, the AASA of residues around the acetylation sites and non-methylation sites have a little difference. The possible reason for limiting the ASA analysis in the acetylation might be that the AASA values of different amino acids are statistical results, and there may be certain differences with the experimental results.

### Using IG to Analyze and Extract Sequences

It is well known that acetylation is catalyzed by conserved enzymatic machinery composed of KATs and KDACs. Chou et al. [51] stated that part of the biochemical preference of the enzyme for a given substrate could be determined by critical neighboring residues that surround the site of action immediately. This pattern of residues along the short span of a protein or polypeptide is known as a short linear motif [52]. And also, according to site-level conservation analysis, Weinert et al. [23] revealed that acetylation sites are highly conserved, significantly more so than phosphorylation sites. These suggest that the catalytic of KATs or KDACs are significantly determined by specificity residues surrounding the acetylation sites. Therefore, it is necessary to carefully examine the position specific amino acid within the sequences of acetylated and non-acetylated proteins and to identify distinctive amino acid enrichment/depletion profiles for acetylated proteins.

**Figure 4:**
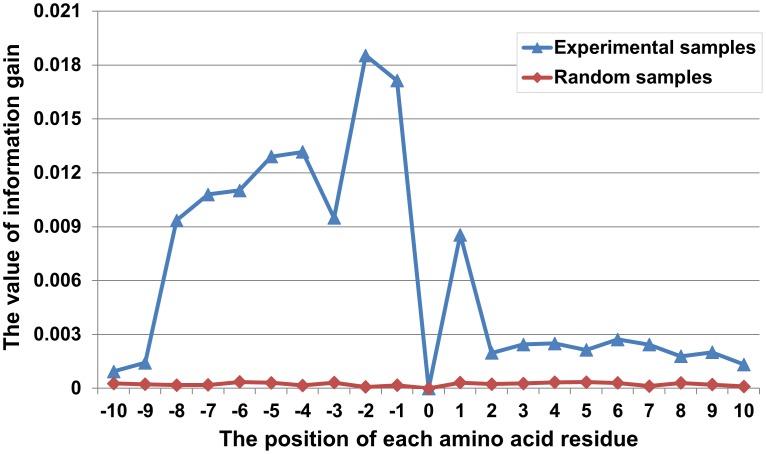
The information gain values at different positions of residues in the sequence fragments.

Based on the measurement of the IG in a large window size (−10∼K∼+10), Figure 4 displays the statistically significant composition of each position of amino acid residues. The surrounding positions that have high values (significant for differentiating the acetylation sites from the non-acetylation sites) are the significant amino acids in the surrounding region. We find that the values in different positions of residues had relatively large changes. Simultaneously, the curves obtained by the experimental acetylation and non-acetylation sequences are clearly above the random curve (created by the random sequence fragments, the number of which is the same as the experimental samples). From this observation, we can draw conclusions that the local acetylation sequences have own unique nature and characteristics and that it can also effectively reflect the differences among the different positions of the amino acid residues. The residues that are closer to the sites did not obtain all of the higher values; in contrast, some of the residues that are far from the acetylated site had higher values. For example, the positions of −8, −7, −6, −5, −4, −3, −2, −1 and +1 have higher values, while compared with other surrounding positions, +5 and +8 have small scores. More interestingly, we can find that the value of the upstream residues generally is higher than that of downstream. It can imply that the KATs more tend to conjugate upstream amino acid residues of the acetylation sites, and this corresponded with the result of Lu’s [53]. In this regard, to improve the prediction performance of acetylation site, we need only choose those locations of amino acids with higher values to rebuild new sequence fragments. Here, we defined the length of new sequence fragment as IG window size. Table 1 displays the different IG window sizes according to the IG values of different positions of amino acid residues. The most appropriate size of IG window was examined in the following work.

**Table 1:** The sizes and positions of IG window.

IG window size	Positions in original 21-mer acetylation sequence fragment
9	−8, −7, −6, −5, −4, −3, −2, −1, +1
11	−8, −7, −6, −5, −4, −3, −2, −1, +1, +4, +6
13	−8, −7, −6, −5, −4, −3, −2, −1, +1, +3, +4, +6, +7
15	−8, −7, −6, −5, −4, −3, −2, −1, +1, +3, +4, +5, +6, +7, +9
17	−8, −7, −6, −5, −4, −3, −2, −1, +1, +2, +3, +4, +5, +6, +7, +8, +9

### Position-specific Prediction of Acetylation Site

To evaluate the performance of PSKAcePred, we chose IG window size as 13 and carried out a 10-fold cross-validation. The predictive performances of models trained with various features for lysine acetylation are shown in Table 2. The predictive results of these models with IG window size 9, 11, 15 and 17 are summarized in Supplementary Table S3–S6. According to statistical comparison of MCC based on the paired Welch’s t-test (see Table S7), the model trained with KNN outperformed that trained with BE and AASA (*P*≤7.83e-10), which is in agreement with the results of above feature analysis. But in general, the models trained with single features could not effectively distinguish acetylation sites from non-acetylation sites. Fortunately, when the model was trained with the combination of BE, KNN and AASA features (BE+KNN+AASA), the performance was remarkably enhanced (*P*≤1.47e-08). The average accuracy, sensitivity, specificity and MCC for acetyllysine were 79.84%, 78.02%, 81.66% and 59.72%, respectively. This result demonstrated that all three types of features contributed to distinguishing between acetylation sites and non-acetylation sites. There was a strong complementary effect among these features. Henceforth, the combination of BE+KNN+AASA was selected as an optimal feature set to improve the predictive model.

**Table 2:** The predictive performance of the models trained with various features with an IG window size of 13.

Trainingfeatures	Accuracy(%)	Sensitivity (%)	Specificity (%)	MCC (%)
BE	68.00±0.17	63.94±0.28	72.06±0.26	36.12±0.33
KNN	74.98±0.39	72.66±0.67	77.31±0.30	50.02±0.78
AASA	65.28±0.13	62.66±0.45	67.90±0.41	30.61±0.26
BE+KNN+AASA	79.84±0.18	78.02±0.20	81.66±0.17	59.72±0.35

Abbreviations: BE, binary encoding; KNN, *K* nearest neighbors; AASA, average accessible surface area. The corresponding measurement was represented as the average value ± standard deviation.

### Determination of the Best IG Window Size

For each acetylation or non-acetylation site, its profile features were taken from a sequence fragment containing several key residues (spatially); thus, it was crucial to confirm the optimal IG window size and to realize its effects on the prediction performance. When using the optimal feature (BE+KNN+AASA), and with the window size changed from 9 to 17 (Table 1), the performance of the models had some differences, as shown in Table 3. The results showed that the IG window size had certain impact on all prediction performance. Based on statistical comparison of MCC (see Table S8), there were significant differences between the prediction model with IG window size of 13 and those of 9, 11, 15, 17 (*P*≤3.01e-07). With the increase of the length of the IG window, the predictive performance was not always increased. When the IG window was too large, much redundant information would be contained, while the IG window was too small, a lot of useful information would lose. The predictive accuracy, sensitivity and MCC reached maximums with the IG window size of 13. Then, based on the computational efficiency and overall performance of the trained models, 13-mer was selected as the best IG window size in the following implementation.

**Table 3:** Prediction performance of the models trained with different IG window sizes.

Performance	9	11	13	15	17
Accuracy (%)	76.69±0.14	77.56±0.23	79.84±0.18	76.97±0.10	76.76±0.21
Sensitivity (%)	75.00±0.35	75.60±0.14	78.02±0.20	75.47±0.19	74.17±0.24
Specificity (%)	78.38±0.35	79.51±0.46	81.66±0.17	78.46±0.12	79.36±0.35
MCC (%)	53.42±0.29	55.15±0.47	59.72±0.35	53.96±0.19	53.60±0.42

The corresponding measurement was represented as the average value ± standard deviation.

### General Prediction of Acetylation Site

To determine the superiority of the IG for optimizing the windows, we construct other seven models by using different general window sizes (where the upstream and downstream amino acids around the central site were continuous and the numbers of them were the same). As shown in Table 4, when using the BE+KNN+AASA feature in 10-fold cross-validation, the prediction performances had significant change with the window size of 9, 11 and 13 (2.72e-06≤*P*≤9.73e-03, see Table S9). When the window size increased to 15, the prediction performances had significant increase (5.17e-08≤*P*≤1.68e-03). However, with the window size continues to increase, the performances did not change significantly (*P*≥0.05). The best prediction performances were obtained when adopted the general window size of 15. However, the average accuracy, sensitivity, specificity and MCC for this best window size were only 76.85%, 74.90%, 78.80% and 53.74%, respectively. Compared with the best result that was obtained by the position-specific prediction method with the IG window size of 13 (Table 3), all of the predictive performances of general prediction method were far inferior to position-specific prediction method, especially for the MCC (reduced by 5.98%, *P* = 1.47e-08). The main reason for this result might be that the KATs are position-specific catalysts that catalyze the acetylation substrate and there are specific conservative residues around the acetylation sites. The IG method could effectively extract those key conservative residues for acetylation prediction.

**Table 4:** The predictive performance of the model trained with optimal feature with general window size.

General window size	Accuracy (%)	Sensitivity (%)	Specificity (%)	MCC (%)
9 (−4∼K∼+4)	73.72±0.25	71.98±0.23	75.47±0.43	47.48±0.51
11 (−5∼K∼+5)	75.42±0.19	73.53±0.31	77.31±0.14	50.87±0.38
13 (−6∼K∼+6)	76.01±0.30	73.52±0.51	78.49±0.46	52.08±0.60
15 (−7∼K∼+7)	76.85±0.19	74.90±0.28	78.80±0.41	53.74±0.39
17 (−8∼K∼+8)	76.82±0.20	74.93±0.20	78.70±0.56	53.67±0.42
19 (−9∼K∼+9)	76.83±0.13	74.55±0.31	79.11±0.18	53.71±0.25
21 (−10∼K∼+10)	76.52±0.09	74.00±0.46	79.04±0.31	53.11±0.16

The corresponding measurement was represented as the average value ± standard deviation.

### Independent Test and Comparisons with Existing Methods

To determine whether the predictive model PSKAcePred is over-fitting for the training data, we applied an independent test set as the benchmark. The test set covered 520 lysine acetylated proteins, which contained 1068 acetylation sites and 15152 non-acetylation sites. None of independent test proteins was included in the training dataset (as described in Data collection and preprocessing). As shown in Table 5, the average accuracy, sensitivity, specificity and MCC for PSKAcePred were 78.79%, 77.34%, 80.24% and 57.61%, respectively. Generally, the prediction model is acceptable and reasonable when the performance in an independent test is just a little lower than those obtained in training test (Table 3 of IG window size 13) [17]. Therefore, the model PSKAcePred is reasonable for predicting lysine acetylation.

**Table 5:** The comparison of predictive performance between our method and other prediction methods on independent test data sets.

Prediction method	Accuracy (%)	Sensitivity (%)	Specificity (%)	MCC (%)
LysAcet	54.43±0.99	56.55±0.00	52.30±1.97	8.87±1.97
EnsemblePail	57.17±0.93	76.12±0.00	38.22±1.86	15.49±1.88
Phosida	64.73±0.44	39.98±0.00	89.47±0.88	33.91±1.20
PSKAcePred	78.79±0.33	77.34±0.00	80.24±0.66	57.61±0.67

The corresponding measurement was represented as the average value ± standard deviation.

In order to further evaluate the prediction performance of the PSKAcePred objectively, we made comparisons with other existing predictors. Here we put our independent test set into three previously developed methods with optimal parameters: LysAcet [19], EnsemblePail [18] and Phosida [15]. The comparisons of predictive performance between our method and other prediction methods are shown in Table 5 and Table S10. It was obvious that PSKAcePred yielded the best performance. The accuracy of PSKAcePred reached 78.79%, which was about 24.36%, 21.62% and 14.06% higher than those in LysAcet, EnsemblePail and Phosida, respectively. Especially, the MCC in PSKAcePred was increased by 48.74%, 42.12% and 23.7% in comparison with the results in LysAcet, EnsemblePail and Phosida, respectively (*P*≤5.46e-10). The training data of these three existing predictors were mainly only from the UniProtKB/Swiss-Prot and our significant improvements can be attributed to the adoption of complete acetylation data not only in UniProtKB/Swiss-Prot but also in CPLA, PhosphoSitePlus, HPRD and SysPTM databases. All of these existing methods are statistical or machine learning based predictors, and they use different sequence features. Compared with these predictors, it is worth mentioning that the formula of the PSKAcePred encoding is much more concise. More importantly, the reasonably good performance of PSKAcePred reflected that the PSKAcePred model can effectively capture the information of critical neighboring residues around lysine acetylation sites.

## Discussion

Protein lysine acetylation has emerged as a key posttranslational modification in cellular regulation [54]. To fully decipher the molecular mechanisms of acetylation-related biological processes, an initial but crucial step is the recognition of acetylated substrates and the corresponding acetylation sites. However, even the advanced laboratory techniques used to analyze and identify acetylation sites, such as mass spectrometry (MS), cannot analyze all types of proteins [14]. For example, the MS method has limited detection and sensitivity capabilities and cannot recover peptides that are acetylated at only low levels. Besides, lysine could be modified only in distinct environment conditions, cell cycle stages, and cell types, and are therefore undetectable in cell extracts we used. Therefore, the prediction of acetylation sites with computational approaches is desirable and necessary. Our prediction model, PSKAcePred, is a promising step toward finding novel acetylation sites, although we did not achieve full prediction capacity.

We performed an in-depth analysis of why this model can obtain such good performance. First, the multiple features were adapted to more comprehensively represent the protein sequences (described in the Results section). Second, the IG was applied to select conservative amino acid residues around the acetylation sites to rebuild the peptides. In the case of protein acetylation, Marmorstein et al. [21], [22] have reviewed that KATs are the catalytic subunits of multisubunit protein complexes that acetylate specific lysine residues on the N-terminal regions of the histone components of chromatin to promote gene activation. Functional data demonstrate that KAT proteins and KAT complexes for a given substrate can be determined by specificity residues surrounding the site [55], [56], and biochemists have focused on identifying those critical neighboring residues that give rise to specific enzyme-substrate interactions. For example, Kim et al. [57] analyzed the differences in the preferences of amino acid residues flanking acetyllysine residues and suggested that the KATs binding motifs showed a positional specificity. The amino acid residues at positions −2, −1, +2 and +4 in relative density maps had obvious high relative abundances. Therefore, they stated that it is highly likely that linear lysine acetylation motifs exist among lysine-acetylated proteins. Additionally, Choudhary et al. [10] used the experimental method to analyze the local sequence context around the acetylation sites and found that amino acids with a bulky side chain were enriched in the −2 and +1 positions, and a propensity for lysine or acetylated lysine at the −4 or +4 position was observed. From the above description, it can be speculated that each position of the individual amino acid residues around acetylation sites have its own inherent characteristics and they play different degree of influence for the happening of acetylation. In this regard, it is reasonable and necessary to extract key amino acid residues from long acetylation substrate for prediction of acetylation sites.

Based on the existing data, we proposed the PSKAcePred for the prediction of acetylation sites. Several issues must be solved in the future researches. (i) We considered the non-annotated lysine residues as non-acetylated sites from the same proteins as that of acetylated sites. Although a large majority of them are non-acetylated sites and we tired our best to reduce the false non-acetylated sites by selecting five negative sets repetitively, some of the non-acetylated sites might be determined to be acetylation sites in the future. (ii) The specific KATs or KDACs of acetylation on a proteome-wide level is largely unknown. The information of validated KATs or KDACs that can be used in acetylation conservation analysis should be applied to supply the support of IG analysis. (iii) With the increase of protein structure data in PDB dataset, the experimental structure information should be contained in extracted feature for acetylation prediction. Many researches have revealed the occurrence of acetylation is related to the secondary structure of a protein [58], [59], [60]. However, when applied the PSIPRED [61], [62], an effective tool for secondary structure prediction, we did not obtain promising result (the result was not list). The main reason for this is probably that there are some differences between the predicted and experimental structure information of acetylation proteins.

Overall, our method presented a new position-specific view to analyze the characteristics of acetylation protein, and considered not only protein sequence information but also evolution similarity of lysine acetylation fragments and physicochemical properties of amino acids. The prediction model achieved a promising performance and outperformed other prediction tools. Feature analyses demonstrate that acetyllysine sites and non-acetyllysine have some significant differences in position specific properties of residues, evolution similarity and average accessible surface area, and the prediction model with multiple features can make full use of the supplementary information from different features to improve classification performance. The detailed feature analysis in this work might help understand the lysine acetylation mechanism and guide the related experimental validation.

## Supporting Information

Table S1 **The detailed information for training set and independent set.**
(DOC)

Table S2 **The cross-validation accuracy of all 544 physicochemical properties.**
(DOC)

Table S3 **The predictive performance of the models trained with various features with an IG window size of 9.**
(DOC)

Table S4 **The predictive performance of the models trained with various features with an IG window size of 11.**
(DOC)

Table S5 **The predictive performance of the models trained with various features with an IG window size of 15.**
(DOC)

Table S6 **The predictive performance of the models trained with various features with an IG window size of 17.**
(DOC)

Table S7 **The MCC of model trained with different features is compared via** ***P*** **-values on the paired Welch’s t-test.** For the entry at row *i*, column *j* of the table, there is statistical difference when P≤0.05, or else there isn’t significantly different.
(DOC)

Table S8 **The MCC of models with different IG window size is compared via** ***P*** **-values on the paired Welch’s t-test.**
(DOC)

Table S9 **The MCC of models with different general window size is compared via** ***P*** **-values on the paired Welch’s t-test.**
(DOC)

Table S10 **The MCC of our method and other prediction methods is compared via** ***P*** **-values on the paired Welch’s t-test.**
(DOC)
