# Supplementary material for: Position-Specific Analysis and Prediction for Protein Lysine Acetylation Based on Multiple Features
Source: PLoS One. 2012 Nov 16;7(11):e49108. doi: 10.1371/journal.pone.0049108 (PMC3500252; doi:10.1371/journal.pone.0049108)
Supplement: Table S1 — The detailed information for training set and independent set. (DOC) [file pone.0049108.s001.doc]

**Table S1.** The detailed information for training set and independent set.

| Dataset | Number of protein | Number of positive set  (All/Less than 30% similarity) | Number of negative set  (All/ Less than 30% similarity) |
| --- | --- | --- | --- |
| Training set | 4682 | 9815/9232 | 217658/131679 |
| Independent set | 520 | 1224/1068 | 24976/15152 |
| In total | 5202 | 11039/10300 | 242634/146831 |
