# Supplementary material for: Position-Specific Analysis and Prediction for Protein Lysine Acetylation Based on Multiple Features
Source: PLoS One. 2012 Nov 16;7(11):e49108. doi: 10.1371/journal.pone.0049108 (PMC3500252; doi:10.1371/journal.pone.0049108)
Supplement: Table S2 — The cross-validation accuracy of all 544 physicochemical properties. (DOC) [file pone.0049108.s002.doc]

**Table S2.** The cross-validation accuracy of all 544 physicochemical properties.

| **NO.** | **AAindex ID** | **Name of feature (References)** | **Accuracy** |
| --- | --- | --- | --- |
| 1 | ANDN920101 | alpha-CH chemical shifts (Andersen et al., 1992) | 53.55828 |
| 2 | ARGP820101 | Hydrophobicity index (Argos et al., 1982) | 55.76798 |
| 3 | ARGP820102 | Signal sequence helical potential (Argos et al., 1982) | 58.41638 |
| 4 | ARGP820103 | Membrane-buried preference parameters (Argos et al., 1982) | 56.9974 |
| 5 | BEGF750101 | Conformational parameter of inner helix (Beghin-Dirkx, 1975) | 53.99155 |
| 6 | BEGF750102 | Conformational parameter of beta-structure (Beghin-Dirkx, 1975) | 54.67938 |
| 7 | BEGF750103 | Conformational parameter of beta-turn (Beghin-Dirkx, 1975) | 52.42093 |
| 8 | BHAR880101 | Average flexibility indices (Bhaskaran-Ponnuswamy, 1988) | 52.90836 |
| 9 | BIGC670101 | Residue volume (Bigelow, 1967) | 55.93046 |
| 10 | BIOV880101 | Information value for accessibility; average fraction 35% (Biou et al., 1988) | 59.6133 |
| 11 | BIOV880102 | Information value for accessibility; average fraction 23% (Biou et al., 1988) | 60.98354 |
| 12 | BROC820101 | Retention coefficient in TFA (Browne et al., 1982) | 54.10529 |
| 13 | BROC820102 | Retention coefficient in HFBA (Browne et al., 1982) | 55.21555 |
| 14 | BULH740101 | Transfer free energy to surface (Bull-Breese, 1974) | 53.737 |
| 15 | BULH740102 | Apparent partial specific volume (Bull-Breese, 1974) | 54.09445 |
| 16 | BUNA790101 | alpha-NH chemical shifts (Bundi-Wuthrich, 1979) | 52.2422 |
| 17 | BUNA790102 | alpha-CH chemical shifts (Bundi-Wuthrich, 1979) | 54.23527 |
| 18 | BUNA790103 | Spin-spin coupling constants 3JHalpha-NH (Bundi-Wuthrich, 1979) | 53.08167 |
| 19 | BURA740101 | Normalized frequency of alpha-helix (Burgess et al., 1974) | 52.48592 |
| 20 | BURA740102 | Normalized frequency of extended structure (Burgess et al., 1974) | 55.71924 |
| 21 | CHAM810101 | Steric parameter (Charton, 1981) | 52.48050 |
| 22 | CHAM820101 | Polarizability parameter (Charton-Charton, 1982) | 54.77145 |
| 23 | CHAM820102 | Free energy of solution in water, kcal/mole (Charton-Charton, 1982) | 53.78575 |
| 24 | CHAM830101 | The Chou-Fasman parameter of the coil conformation (Charton-Charton, 1983) | 53.47704 |
| 25 | CHAM830102 | A parameter defined from the residuals obtained from the best correlation of the Chou-Fasman parameter of beta-sheet (Charton-Charton, 1983) | 52.52383 |
| 26 | CHAM830103 | The number of atoms in the side chain labelled 1+1 (Charton-Charton, 1983) | 52.81088 |
| 27 | CHAM830104 | The number of atoms in the side chain labelled 2+1 (Charton-Charton, 1983) | 54.32734 |
| 28 | CHAM830105 | The number of atoms in the side chain labelled 3+1 (Charton-Charton, 1983) | 56.18501 |
| 29 | CHAM830106 | The number of bonds in the longest chain (Charton-Charton, 1983) | 57.84229 |
| 30 | CHAM830107 | A parameter of charge transfer capability (Charton-Charton, 1983) | 51.42439 |
| 31 | CHAM830108 | A parameter of charge transfer donor capability (Charton-Charton, 1983) | 55.69216 |
| 32 | CHOC750101 | Average volume of buried residue (Chothia, 1975) | 54.47899 |
| 33 | CHOC760101 | Residue accessible surface area in tripeptide (Chothia, 1976) | 56.60745 |
| 34 | CHOC760102 | Residue accessible surface area in folded protein (Chothia, 1976) | 65.14840 |
| 35 | CHOC760103 | Proportion of residues 95% buried (Chothia, 1976) | 59.68912 |
| 36 | CHOC760104 | Proportion of residues 100% buried (Chothia, 1976) | 58.85507 |
| 37 | CHOP780101 | Normalized frequency of beta-turn (Chou-Fasman, 1978a) | 54.35984 |
| 38 | CHOP780201 | Normalized frequency of alpha-helix (Chou-Fasman, 1978b) | 52.43176 |
| 39 | CHOP780202 | Normalized frequency of beta-sheet (Chou-Fasman, 1978b) | 54.07821 |
| 40 | CHOP780203 | Normalized frequency of beta-turn (Chou-Fasman, 1978b) | 54.77145 |
| 41 | CHOP780204 | Normalized frequency of N-terminal helix (Chou-Fasman, 1978b) | 53.60702 |
| 42 | CHOP780205 | Normalized frequency of C-terminal helix (Chou-Fasman, 1978b) | 63.23657 |
| 43 | CHOP780206 | Normalized frequency of N-terminal non helical region (Chou-Fasman, 1978b) | 53.66118 |
| 44 | CHOP780207 | Normalized frequency of C-terminal non helical region (Chou-Fasman, 1978b) | 56.37998 |
| 45 | CHOP780208 | Normalized frequency of N-terminal beta-sheet (Chou-Fasman, 1978b) | 55.63258 |
| 46 | CHOP780209 | Normalized frequency of C-terminal beta-sheet (Chou-Fasman, 1978b) | 52.82171 |
| 47 | CHOP780210 | Normalized frequency of N-terminal non beta region (Chou-Fasman, 1978b) | 54.20819 |
| 48 | CHOP780211 | Normalized frequency of C-terminal non beta region (Chou-Fasman, 1978b) | 53.11417 |
| 49 | CHOP780212 | Frequency of the 1st residue in turn (Chou-Fasman, 1978b) | 54.38692 |
| 50 | CHOP780213 | Frequency of the 2nd residue in turn (Chou-Fasman, 1978b) | 57.19779 |
| 51 | CHOP780214 | Frequency of the 3rd residue in turn (Chou-Fasman, 1978b) | 52.67548 |
| 52 | CHOP780215 | Frequency of the 4th residue in turn (Chou-Fasman, 1978b) | 54.50065 |
| 53 | CHOP780216 | Normalized frequency of the 2nd and 3rd residues in turn (Chou-Fasman, 1978b) | 55.84380 |
| 54 | CIDH920101 | Normalized hydrophobicity scales for alpha-proteins (Cid et al., 1992) | 54.95017 |
| 55 | CIDH920102 | Normalized hydrophobicity scales for beta-proteins (Cid et al., 1992) | 54.95017 |
| 56 | CIDH920103 | Normalized hydrophobicity scales for alpha+beta-proteins (Cid et al., 1992) | 54.45191 |
| 57 | CIDH920104 | Normalized hydrophobicity scales for alpha/beta-proteins (Cid et al., 1992) | 54.95559 |
| 58 | CIDH920105 | Normalized average hydrophobicity scales (Cid et al., 1992) | 55.41594 |
| 59 | COHE430101 | Partial specific volume (Cohn-Edsall, 1943) | 54.82019 |
| 60 | CRAJ730101 | Normalized frequency of middle helix (Crawford et al., 1973) | 52.93544 |
| 61 | CRAJ730102 | Normalized frequency of beta-sheet (Crawford et al., 1973) | 55.18306 |
| 62 | CRAJ730103 | Normalized frequency of turn (Crawford et al., 1973) | 53.01127 |
| 63 | DAWD720101 | Size (Dawson, 1972) | 60.78315 |
| 64 | DAYM780101 | Amino acid composition (Dayhoff et al., 1978a) | 54.27318 |
| 65 | DAYM780201 | Relative mutability (Dayhoff et al., 1978b) | 55.25888 |
| 66 | DESM900101 | Membrane preference for cytochrome b: MPH89 (Degli Esposti et al., 1990) | 57.18696 |
| 67 | DESM900102 | Average membrane preference: AMP07 (Degli Esposti et al., 1990) | 55.87630 |
| 68 | EISD840101 | Consensus normalized hydrophobicity scale (Eisenberg, 1984) | 59.46166 |
| 69 | EISD860101 | Solvation free energy (Eisenberg-McLachlan, 1986) | 56.59662 |
| 70 | EISD860102 | Atom-based hydrophobic moment (Eisenberg-McLachlan, 1986) | 60.75607 |
| 71 | EISD860103 | Direction of hydrophobic moment (Eisenberg-McLachlan, 1986) | 55.91963 |
| 72 | FASG760101 | Molecular weight (Fasman, 1976) | 54.09445 |
| 73 | FASG760102 | Melting point (Fasman, 1976) | 53.94281 |
| 74 | FASG760103 | Optical rotation (Fasman, 1976) | 57.91269 |
| 75 | FASG760104 | pK-N (Fasman, 1976) | 52.49133 |
| 76 | FASG760105 | pK-C (Fasman, 1976) | 53.76950 |
| 77 | FAUJ830101 | Hydrophobic parameter pi (Fauchere-Pliska, 1983) | 59.81911 |
| 78 | FAUJ880101 | Graph shape index (Fauchere et al., 1988) | 54.04029 |
| 79 | FAUJ880102 | Smoothed upsilon steric parameter (Fauchere et al., 1988) | 51.28899 |
| 80 | FAUJ880103 | Normalized van der Waals volume (Fauchere et al., 1988) | 55.12890 |
| 81 | FAUJ880104 | STERIMOL length of the side chain (Fauchere et al., 1988) | 59.86785 |
| 82 | FAUJ880105 | STERIMOL minimum width of the side chain (Fauchere et al., 1988) | 53.86698 |
| 83 | FAUJ880106 | STERIMOL maximum width of the side chain (Fauchere et al., 1988) | 55.04224 |
| 84 | FAUJ880107 | N.m.r. chemical shift of alpha-carbon (Fauchere et al., 1988) | 54.76603 |
| 85 | FAUJ880108 | Localized electrical effect (Fauchere et al., 1988) | 54.69562 |
| 86 | FAUJ880109 | Number of hydrogen bond donors (Fauchere et al., 1988) | 57.73938 |
| 87 | FAUJ880110 | Number of full nonbonding orbitals (Fauchere et al., 1988) | 53.48787 |
| 88 | FAUJ880111 | Positive charge (Fauchere et al., 1988) | 62.51083 |
| 89 | FAUJ880112 | Negative charge (Fauchere et al., 1988) | 50.90988 |
| 90 | FAUJ880113 | pK-a(RCOOH) (Fauchere et al., 1988) | 52.92461 |
| 91 | FINA770101 | Helix-coil equilibrium constant (Finkelstein-Ptitsyn, 1977) | 54.43566 |
| 92 | FINA910101 | Helix initiation parameter at posision i-1 (Finkelstein et al., 1991) | 56.51538 |
| 93 | FINA910102 | Helix initiation parameter at posision i,i+1,i+2 (Finkelstein et al., 1991) | 52.92461 |
| 94 | FINA910103 | Helix termination parameter at posision j-2,j-1,j (Finkelstein et al., 1991) | 62.25628 |
| 95 | FINA910104 | Helix termination parameter at posision j+1 (Finkelstein et al., 1991) | 64.18436 |
| 96 | GARJ730101 | Partition coefficient (Garel et al., 1973) | 55.18847 |
| 97 | GEIM800101 | Alpha-helix indices (Geisow-Roberts, 1980) | 53.87240 |
| 98 | GEIM800102 | Alpha-helix indices for alpha-proteins (Geisow-Roberts, 1980) | 53.13583 |
| 99 | GEIM800103 | Alpha-helix indices for beta-proteins (Geisow-Roberts, 1980) | 51.89558 |
| 100 | GEIM800104 | Alpha-helix indices for alpha/beta-proteins (Geisow-Roberts, 1980) | 52.75130 |
| 101 | GEIM800105 | Beta-strand indices (Geisow-Roberts, 1980) | 53.65035 |
| 102 | GEIM800106 | Beta-strand indices for beta-proteins (Geisow-Roberts, 1980) | 53.81824 |
| 103 | GEIM800107 | Beta-strand indices for alpha/beta-proteins (Geisow-Roberts, 1980) | 54.25152 |
| 104 | GEIM800108 | Aperiodic indices (Geisow-Roberts, 1980) | 51.52188 |
| 105 | GEIM800109 | Aperiodic indices for alpha-proteins (Geisow-Roberts, 1980) | 53.09792 |
| 106 | GEIM800110 | Aperiodic indices for beta-proteins (Geisow-Roberts, 1980) | 54.97725 |
| 107 | GEIM800111 | Aperiodic indices for alpha/beta-proteins (Geisow-Roberts, 1980) | 53.10334 |
| 108 | GOLD730101 | Hydrophobicity factor (Goldsack-Chalifoux, 1973) | 56.72660 |
| 109 | GOLD730102 | Residue volume (Goldsack-Chalifoux, 1973) | 56.25542 |
| 110 | GRAR740101 | Composition (Grantham, 1974) | 54.07279 |
| 111 | GRAR740102 | Polarity (Grantham, 1974) | 55.67591 |
| 112 | GRAR740103 | Volume (Grantham, 1974) | 56.34207 |
| 113 | GUYH850101 | Partition energy (Guy, 1985) | 59.84077 |
| 114 | HOPA770101 | Hydration number (Hopfinger, 1971), Cited by Charton-Charton (1982) | 57.49025 |
| 115 | HOPT810101 | Hydrophilicity value (Hopp-Woods, 1981) | 59.29918 |
| 116 | HUTJ700101 | Heat capacity (Hutchens, 1970) | 58.27015 |
| 117 | HUTJ700102 | Absolute entropy (Hutchens, 1970) | 60.33362 |
| 118 | HUTJ700103 | Entropy of formation (Hutchens, 1970) | 61.15685 |
| 119 | ISOY800101 | Normalized relative frequency of alpha-helix (Isogai et al., 1980) | 52.58882 |
| 120 | ISOY800102 | Normalized relative frequency of extended structure (Isogai et al., 1980) | 56.75910 |
| 121 | ISOY800103 | Normalized relative frequency of bend (Isogai et al., 1980) | 53.67201 |
| 122 | ISOY800104 | Normalized relative frequency of bend R (Isogai et al., 1980) | 57.48484 |
| 123 | ISOY800105 | Normalized relative frequency of bend S (Isogai et al., 1980) | 52.62673 |
| 124 | ISOY800106 | Normalized relative frequency of helix end (Isogai et al., 1980) | 52.58341 |
| 125 | ISOY800107 | Normalized relative frequency of double bend (Isogai et al., 1980) | 52.83795 |
| 126 | ISOY800108 | Normalized relative frequency of coil (Isogai et al., 1980) | 54.09445 |
| 127 | JANJ780101 | Average accessible surface area (Janin et al., 1978) | 65.40836 |
| 128 | JANJ780102 | Percentage of buried residues (Janin et al., 1978) | 61.11352 |
| 129 | JANJ780103 | Percentage of exposed residues (Janin et al., 1978) | 64.05481 |
| 130 | JANJ790101 | Ratio of buried and accessible molar fractions (Janin, 1979) | 56.32582 |
| 131 | JANJ790102 | Transfer free energy (Janin, 1979) | 64.66833 |
| 132 | JOND750101 | Hydrophobicity (Jones, 1975) | 55.74632 |
| 133 | JOND750102 | pK (-COOH) (Jones, 1975) | 53.88865 |
| 134 | JOND920101 | Relative frequency of occurrence (Jones et al., 1992) | 54.04571 |
| 135 | JOND920102 | Relative mutability (Jones et al., 1992) | 55.13432 |
| 136 | JUKT750101 | Amino acid distribution (Jukes et al., 1975) | 54.22444 |
| 137 | JUNJ780101 | Sequence frequency (Jungck, 1978) | 55.57301 |
| 138 | KANM800101 | Average relative probability of helix (Kanehisa-Tsong, 1980) | 53.02751 |
| 139 | KANM800102 | Average relative probability of beta-sheet (Kanehisa-Tsong, 1980) | 54.21360 |
| 140 | KANM800103 | Average relative probability of inner helix (Kanehisa-Tsong, 1980) | 52.68631 |
| 141 | KANM800104 | Average relative probability of inner beta-sheet (Kanehisa-Tsong, 1980) | 54.02405 |
| 142 | KARP850101 | Flexibility parameter for no rigid neighbors (Karplus-Schulz, 1985) | 55.12348 |
| 143 | KARP850102 | Flexibility parameter for one rigid neighbor (Karplus-Schulz, 1985) | 56.92699 |
| 144 | KARP850103 | Flexibility parameter for two rigid neighbors (Karplus-Schulz, 1985) | 65.10745 |
| 145 | KHAG800101 | The Kerr-constant increments (Khanarian-Moore, 1980) | 56.48289 |
| 146 | KLEP840101 | Net charge (Klein et al., 1984) | 64.67179 |
| 147 | KRIW710101 | Side chain interaction parameter (Krigbaum-Rubin, 1971) | 61.77426 |
| 148 | KRIW790101 | Side chain interaction parameter (Krigbaum-Komoriya, 1979) | 63.11200 |
| 149 | KRIW790102 | Fraction of site occupied by water (Krigbaum-Komoriya, 1979) | 64.30741 |
| 150 | KRIW790103 | Side chain volume (Krigbaum-Komoriya, 1979) | 56.06044 |
| 151 | KYTJ820101 | Hydropathy index (Kyte-Doolittle, 1982) | 57.07322 |
| 152 | LAWE840101 | Transfer free energy, CHP/water (Lawson et al., 1984) | 53.93739 |
| 153 | LEVM760101 | Hydrophobic parameter (Levitt, 1976) | 60.35529 |
| 154 | LEVM760102 | Distance between C-alpha and centroid of side chain (Levitt, 1976) | 57.37652 |
| 155 | LEVM760103 | Side chain angle theta(AAR) (Levitt, 1976) | 53.76408 |
| 156 | LEVM760104 | Side chain torsion angle phi(AAAR) (Levitt, 1976) | 53.09792 |
| 157 | LEVM760105 | Radius of gyration of side chain (Levitt, 1976) | 59.81911 |
| 158 | LEVM760106 | van der Waals parameter R0 (Levitt, 1976) | 53.73159 |
| 159 | LEVM760107 | van der Waals parameter epsilon (Levitt, 1976) | 54.37067 |
| 160 | LEVM780101 | Normalized frequency of alpha-helix, with weights (Levitt, 1978) | 52.12305 |
| 161 | LEVM780102 | Normalized frequency of beta-sheet, with weights (Levitt, 1978) | 53.95906 |
| 162 | LEVM780103 | Normalized frequency of reverse turn, with weights (Levitt, 1978) | 52.96252 |
| 163 | LEVM780104 | Normalized frequency of alpha-helix, unweighted (Levitt, 1978) | 53.23332 |
| 164 | LEVM780105 | Normalized frequency of beta-sheet, unweighted (Levitt, 1978) | 54.63063 |
| 165 | LEVM780106 | Normalized frequency of reverse turn, unweighted (Levitt, 1978) | 53.29831 |
| 166 | LEWP710101 | Frequency of occurrence in beta-bends (Lewis et al., 1971) | 52.89753 |
| 167 | LIFS790101 | Conformational preference for all beta-strands (Lifson-Sander, 1979) | 53.93198 |
| 168 | LIFS790102 | Conformational preference for parallel beta-strands (Lifson-Sander, 1979) | 52.74047 |
| 169 | LIFS790103 | Conformational preference for antiparallel beta-strands (Lifson-Sander, 1979) | 54.62522 |
| 170 | MANP780101 | Average surrounding hydrophobicity (Manavalan-Ponnuswamy, 1978) | 54.42483 |
| 171 | MAXF760101 | Normalized frequency of alpha-helix (Maxfield-Scheraga, 1976) | 52.98960 |
| 172 | MAXF760102 | Normalized frequency of extended structure (Maxfield-Scheraga, 1976) | 55.39428 |
| 173 | MAXF760103 | Normalized frequency of zeta R (Maxfield-Scheraga, 1976) | 51.70602 |
| 174 | MAXF760104 | Normalized frequency of left-handed alpha-helix (Maxfield-Scheraga, 1976) | 56.57496 |
| 175 | MAXF760105 | Normalized frequency of zeta L (Maxfield-Scheraga, 1976) | 57.12197 |
| 176 | MAXF760106 | Normalized frequency of alpha region (Maxfield-Scheraga, 1976) | 52.98960 |
| 177 | MCMT640101 | Refractivity (McMeekin et al., 1964), Cited by Jones (1975) | 53.29289 |
| 178 | MEEJ800101 | Retention coefficient in HPLC, pH7.4 (Meek, 1980) | 54.41941 |
| 179 | MEEJ800102 | Retention coefficient in HPLC, pH2.1 (Meek, 1980) | 55.23180 |
| 180 | MEEJ810101 | Retention coefficient in NaClO4 (Meek-Rossetti, 1981) | 54.67938 |
| 181 | MEEJ810102 | Retention coefficient in NaH2PO4 (Meek-Rossetti, 1981) | 56.72119 |
| 182 | MEIH800101 | Average reduced distance for C-alpha (Meirovitch et al., 1980) | 58.21599 |
| 183 | MEIH800102 | Average reduced distance for side chain (Meirovitch et al., 1980) | 63.12283 |
| 184 | MEIH800103 | Average side chain orientation angle (Meirovitch et al., 1980) | 57.97227 |
| 185 | MIYS850101 | Effective partition energy (Miyazawa-Jernigan, 1985) | 60.27946 |
| 186 | NAGK730101 | Normalized frequency of alpha-helix (Nagano, 1973) | 53.54744 |
| 187 | NAGK730102 | Normalized frequency of bata-structure (Nagano, 1973) | 55.35637 |
| 188 | NAGK730103 | Normalized frequency of coil (Nagano, 1973) | 52.90295 |
| 189 | NAKH900101 | AA composition of total proteins (Nakashima et al., 1990) | 54.34359 |
| 190 | NAKH900102 | SD of AA composition of total proteins (Nakashima et al., 1990) | 56.69952 |
| 191 | NAKH900103 | AA composition of mt-proteins (Nakashima et al., 1990) | 54.72812 |
| 192 | NAKH900104 | Normalized composition of mt-proteins (Nakashima et al., 1990) | 56.10377 |
| 193 | NAKH900105 | AA composition of mt-proteins from animal (Nakashima et al., 1990) | 55.64341 |
| 194 | NAKH900106 | Normalized composition from animal (Nakashima et al., 1990) | 57.01906 |
| 195 | NAKH900107 | AA composition of mt-proteins from fungi and plant (Nakashima et al., 1990) | 54.61438 |
| 196 | NAKH900108 | Normalized composition from fungi and plant (Nakashima et al., 1990) | 53.62327 |
| 197 | NAKH900109 | AA composition of membrane proteins (Nakashima et al., 1990) | 52.79463 |
| 198 | NAKH900110 | Normalized composition of membrane proteins (Nakashima et al., 1990) | 59.94367 |
| 199 | NAKH900111 | Transmembrane regions of non-mt-proteins (Nakashima et al., 1990) | 54.46274 |
| 200 | NAKH900112 | Transmembrane regions of mt-proteins (Nakashima et al., 1990) | 56.04961 |
| 201 | NAKH900113 | Ratio of average and computed composition (Nakashima et al., 1990) | 54.82561 |
| 202 | NAKH920101 | AA composition of CYT of single-spanning proteins (Nakashima-Nishikawa, 1992) | 55.63800 |
| 203 | NAKH920102 | AA composition of CYT2 of single-spanning proteins (Nakashima-Nishikawa, 1992) | 53.96989 |
| 204 | NAKH920103 | AA composition of EXT of single-spanning proteins (Nakashima-Nishikawa, 1992) | 55.71382 |
| 205 | NAKH920104 | AA composition of EXT2 of single-spanning proteins (Nakashima-Nishikawa, 1992) | 54.44107 |
| 206 | NAKH920105 | AA composition of MEM of single-spanning proteins (Nakashima-Nishikawa, 1992) | 54.70104 |
| 207 | NAKH920106 | AA composition of CYT of multi-spanning proteins (Nakashima-Nishikawa, 1992) | 56.93241 |
| 208 | NAKH920107 | AA composition of EXT of multi-spanning proteins (Nakashima-Nishikawa, 1992) | 52.22595 |
| 209 | NAKH920108 | AA composition of MEM of multi-spanning proteins (Nakashima-Nishikawa, 1992) | 55.59467 |
| 210 | NISK800101 | 8 A contact number (Nishikawa-Ooi, 1980) | 56.22834 |
| 211 | NISK860101 | 14 A contact number (Nishikawa-Ooi, 1986) | 56.22292 |
| 212 | NOZY710101 | Transfer energy, organic solvent/water (Nozaki-Tanford, 1971) | 54.19736 |
| 213 | OOBM770101 | Average non-bonded energy per atom (Oobatake-Ooi, 1977) | 63.05243 |
| 214 | OOBM770102 | Short and medium range non-bonded energy per atom (Oobatake-Ooi, 1977) | 57.75022 |
| 215 | OOBM770103 | Long range non-bonded energy per atom (Oobatake-Ooi, 1977) | 59.35334 |
| 216 | OOBM770104 | Average non-bonded energy per residue (Oobatake-Ooi, 1977) | 53.91031 |
| 217 | OOBM770105 | Short and medium range non-bonded energy per residue (Oobatake-Ooi, 1977) | 52.78921 |
| 218 | OOBM850101 | Optimized beta-structure-coil equilibrium constant (Oobatake et al., 1985) | 51.98765 |
| 219 | OOBM850102 | Optimized propensity to form reverse turn (Oobatake et al., 1985) | 53.19541 |
| 220 | OOBM850103 | Optimized transfer energy parameter (Oobatake et al., 1985) | 57.35485 |
| 221 | OOBM850104 | Optimized average non-bonded energy per atom (Oobatake et al., 1985) | 56.46122 |
| 222 | OOBM850105 | Optimized side chain interaction parameter (Oobatake et al., 1985) | 64.61222 |
| 223 | PALJ810101 | Normalized frequency of alpha-helix from LG (Palau et al., 1981) | 53.21166 |
| 224 | PALJ810102 | Normalized frequency of alpha-helix from CF (Palau et al., 1981) | 52.64840 |
| 225 | PALJ810103 | Normalized frequency of beta-sheet from LG (Palau et al., 1981) | 54.38150 |
| 226 | PALJ810104 | Normalized frequency of beta-sheet from CF (Palau et al., 1981) | 53.77491 |
| 227 | PALJ810105 | Normalized frequency of turn from LG (Palau et al., 1981) | 53.40121 |
| 228 | PALJ810106 | Normalized frequency of turn from CF (Palau et al., 1981) | 55.17223 |
| 229 | PALJ810107 | Normalized frequency of alpha-helix in all-alpha class (Palau et al., 1981) | 52.86503 |
| 230 | PALJ810108 | Normalized frequency of alpha-helix in alpha+beta class (Palau et al., 1981) | 51.84142 |
| 231 | PALJ810109 | Normalized frequency of alpha-helix in alpha/beta class (Palau et al., 1981) | 52.91919 |
| 232 | PALJ810110 | Normalized frequency of beta-sheet in all-beta class (Palau et al., 1981) | 53.02751 |
| 233 | PALJ810111 | Normalized frequency of beta-sheet in alpha+beta class (Palau et al., 1981) | 53.49328 |
| 234 | PALJ810112 | Normalized frequency of beta-sheet in alpha/beta class (Palau et al., 1981) | 53.74242 |
| 235 | PALJ810113 | Normalized frequency of turn in all-alpha class (Palau et al., 1981) | 54.54398 |
| 236 | PALJ810114 | Normalized frequency of turn in all-beta class (Palau et al., 1981) | 52.82171 |
| 237 | PALJ810115 | Normalized frequency of turn in alpha+beta class (Palau et al., 1981) | 55.16681 |
| 238 | PALJ810116 | Normalized frequency of turn in alpha/beta class (Palau et al., 1981) | 53.39580 |
| 239 | PARJ860101 | HPLC parameter (Parker et al., 1986) | 54.28943 |
| 240 | PLIV810101 | Partition coefficient (Pliska et al., 1981) | 57.15988 |
| 241 | PONP800101 | Surrounding hydrophobicity in folded form (Ponnuswamy et al., 1980) | 55.96837 |
| 242 | PONP800102 | Average gain in surrounding hydrophobicity (Ponnuswamy et al., 1980) | 58.84424 |
| 243 | PONP800103 | Average gain ratio in surrounding hydrophobicity (Ponnuswamy et al., 1980) | 61.21101 |
| 244 | PONP800104 | Surrounding hydrophobicity in alpha-helix (Ponnuswamy et al., 1980) | 52.05806 |
| 245 | PONP800105 | Surrounding hydrophobicity in beta-sheet (Ponnuswamy et al., 1980) | 54.08904 |
| 246 | PONP800106 | Surrounding hydrophobicity in turn (Ponnuswamy et al., 1980) | 61.28141 |
| 247 | PONP800107 | Accessibility reduction ratio (Ponnuswamy et al., 1980) | 57.31153 |
| 248 | PONP800108 | Average number of surrounding residues (Ponnuswamy et al., 1980) | 57.20862 |
| 249 | PRAM820101 | Intercept in regression analysis (Prabhakaran-Ponnuswamy, 1982) | 64.83969 |
| 250 | PRAM820102 | Slope in regression analysis x 1.0E1 (Prabhakaran-Ponnuswamy, 1982) | 63.22574 |
| 251 | PRAM820103 | Correlation coefficient in regression analysis (Prabhakaran-Ponnuswamy, 1982) | 57.17071 |
| 252 | PRAM900101 | Hydrophobicity (Prabhakaran, 1990) | 58.32431 |
| 253 | PRAM900102 | Relative frequency in alpha-helix (Prabhakaran, 1990) | 52.12305 |
| 254 | PRAM900103 | Relative frequency in beta-sheet (Prabhakaran, 1990) | 53.95906 |
| 255 | PRAM900104 | Relative frequency in reverse-turn (Prabhakaran, 1990) | 53.07084 |
| 256 | PTIO830101 | Helix-coil equilibrium constant (Ptitsyn-Finkelstein, 1983) | 52.05806 |
| 257 | PTIO830102 | Beta-coil equilibrium constant (Ptitsyn-Finkelstein, 1983) | 54.80936 |
| 258 | QIAN880101 | Weights for alpha-helix at the window position of -6 (Qian-Sejnowski, 1988) | 63.19562 |
| 259 | QIAN880102 | Weights for alpha-helix at the window position of -5 (Qian-Sejnowski, 1988) | 54.28401 |
| 260 | QIAN880103 | Weights for alpha-helix at the window position of -4 (Qian-Sejnowski, 1988) | 51.89558 |
| 261 | QIAN880104 | Weights for alpha-helix at the window position of -3 (Qian-Sejnowski, 1988) | 54.02946 |
| 262 | QIAN880105 | Weights for alpha-helix at the window position of -2 (Qian-Sejnowski, 1988) | 51.29441 |
| 263 | QIAN880106 | Weights for alpha-helix at the window position of -1 (Qian-Sejnowski, 1988) | 51.62478 |
| 264 | QIAN880107 | Weights for alpha-helix at the window position of 0 (Qian-Sejnowski, 1988) | 52.32344 |
| 265 | QIAN880108 | Weights for alpha-helix at the window position of 1 (Qian-Sejnowski, 1988) | 51.77101 |
| 266 | QIAN880109 | Weights for alpha-helix at the window position of 2 (Qian-Sejnowski, 1988) | 53.59077 |
| 267 | QIAN880110 | Weights for alpha-helix at the window position of 3 (Qian-Sejnowski, 1988) | 54.80394 |
| 268 | QIAN880111 | Weights for alpha-helix at the window position of 4 (Qian-Sejnowski, 1988) | 54.06737 |
| 269 | QIAN880112 | Weights for alpha-helix at the window position of 5 (Qian-Sejnowski, 1988) | 55.35095 |
| 270 | QIAN880113 | Weights for alpha-helix at the window position of 6 (Qian-Sejnowski, 1988) | 62.35377 |
| 271 | QIAN880114 | Weights for beta-sheet at the window position of -6 (Qian-Sejnowski, 1988) | 55.37262 |
| 272 | QIAN880115 | Weights for beta-sheet at the window position of -5 (Qian-Sejnowski, 1988) | 54.29484 |
| 273 | QIAN880116 | Weights for beta-sheet at the window position of -4 (Qian-Sejnowski, 1988) | 54.21902 |
| 274 | QIAN880117 | Weights for beta-sheet at the window position of -3 (Qian-Sejnowski, 1988) | 54.92309 |
| 275 | QIAN880118 | Weights for beta-sheet at the window position of -2 (Qian-Sejnowski, 1988) | 52.51300 |
| 276 | QIAN880119 | Weights for beta-sheet at the window position of -1 (Qian-Sejnowski, 1988) | 54.84727 |
| 277 | QIAN880120 | Weights for beta-sheet at the window position of 0 (Qian-Sejnowski, 1988) | 54.26776 |
| 278 | QIAN880121 | Weights for beta-sheet at the window position of 1 (Qian-Sejnowski, 1988) | 54.77686 |
| 279 | QIAN880122 | Weights for beta-sheet at the window position of 2 (Qian-Sejnowski, 1988) | 55.41594 |
| 280 | QIAN880123 | Weights for beta-sheet at the window position of 3 (Qian-Sejnowski, 1988) | 54.00238 |
| 281 | QIAN880124 | Weights for beta-sheet at the window position of 4 (Qian-Sejnowski, 1988) | 56.67786 |
| 282 | QIAN880125 | Weights for beta-sheet at the window position of 5 (Qian-Sejnowski, 1988) | 58.05351 |
| 283 | QIAN880126 | Weights for beta-sheet at the window position of 6 (Qian-Sejnowski, 1988) | 55.55676 |
| 284 | QIAN880127 | Weights for coil at the window position of -6 (Qian-Sejnowski, 1988) | 64.57127 |
| 285 | QIAN880128 | Weights for coil at the window position of -5 (Qian-Sejnowski, 1988) | 53.44996 |
| 286 | QIAN880129 | Weights for coil at the window position of -4 (Qian-Sejnowski, 1988) | 57.73938 |
| 287 | QIAN880130 | Weights for coil at the window position of -3 (Qian-Sejnowski, 1988) | 56.01711 |
| 288 | QIAN880131 | Weights for coil at the window position of -2 (Qian-Sejnowski, 1988) | 55.45386 |
| 289 | QIAN880132 | Weights for coil at the window position of -1 (Qian-Sejnowski, 1988) | 54.43566 |
| 290 | QIAN880133 | Weights for coil at the window position of 0 (Qian-Sejnowski, 1988) | 53.16291 |
| 291 | QIAN880134 | Weights for coil at the window position of 1 (Qian-Sejnowski, 1988) | 54.66854 |
| 292 | QIAN880135 | Weights for coil at the window position of 2 (Qian-Sejnowski, 1988) | 53.25498 |
| 293 | QIAN880136 | Weights for coil at the window position of 3 (Qian-Sejnowski, 1988) | 52.85420 |
| 294 | QIAN880137 | Weights for coil at the window position of 4 (Qian-Sejnowski, 1988) | 52.49133 |
| 295 | QIAN880138 | Weights for coil at the window position of 5 (Qian-Sejnowski, 1988) | 54.96101 |
| 296 | QIAN880139 | Weights for coil at the window position of 6 (Qian-Sejnowski, 1988) | 56.88367 |
| 297 | RACS770101 | Average reduced distance for C-alpha (Rackovsky-Scheraga, 1977) | 54.41399 |
| 298 | RACS770102 | Average reduced distance for side chain (Rackovsky-Scheraga, 1977) | 62.94952 |
| 299 | RACS770103 | Side chain orientational preference (Rackovsky-Scheraga, 1977) | 60.98354 |
| 300 | RACS820101 | Average relative fractional occurrence in A0(i) (Rackovsky-Scheraga, 1982) | 54.61980 |
| 301 | RACS820102 | Average relative fractional occurrence in AR(i) (Rackovsky-Scheraga, 1982) | 51.40273 |
| 302 | RACS820103 | Average relative fractional occurrence in AL(i) (Rackovsky-Scheraga, 1982) | 58.04268 |
| 303 | RACS820104 | Average relative fractional occurrence in EL(i) (Rackovsky-Scheraga, 1982) | 55.67591 |
| 304 | RACS820105 | Average relative fractional occurrence in E0(i) (Rackovsky-Scheraga, 1982) | 58.55178 |
| 305 | RACS820106 | Average relative fractional occurrence in ER(i) (Rackovsky-Scheraga, 1982) | 54.88518 |
| 306 | RACS820107 | Average relative fractional occurrence in A0(i-1) (Rackovsky-Scheraga, 1982) | 53.28748 |
| 307 | RACS820108 | Average relative fractional occurrence in AR(i-1) (Rackovsky-Scheraga, 1982) | 53.16833 |
| 308 | RACS820109 | Average relative fractional occurrence in AL(i-1) (Rackovsky-Scheraga, 1982) | 53.63410 |
| 309 | RACS820110 | Average relative fractional occurrence in EL(i-1) (Rackovsky-Scheraga, 1982) | 50.82322 |
| 310 | RACS820111 | Average relative fractional occurrence in E0(i-1) (Rackovsky-Scheraga, 1982) | 53.36330 |
| 311 | RACS820112 | Average relative fractional occurrence in ER(i-1) (Rackovsky-Scheraga, 1982) | 56.02795 |
| 312 | RACS820113 | Value of theta(i) (Rackovsky-Scheraga, 1982) | 51.85225 |
| 313 | RACS820114 | Value of theta(i-1) (Rackovsky-Scheraga, 1982) | 54.80394 |
| 314 | RADA880101 | Transfer free energy from chx to wat (Radzicka-Wolfenden, 1988) | 55.99003 |
| 315 | RADA880102 | Transfer free energy from oct to wat (Radzicka-Wolfenden, 1988) | 54.69021 |
| 316 | RADA880103 | Transfer free energy from vap to chx (Radzicka-Wolfenden, 1988) | 53.86698 |
| 317 | RADA880104 | Transfer free energy from chx to oct (Radzicka-Wolfenden, 1988) | 56.2175 |
| 318 | RADA880105 | Transfer free energy from vap to oct (Radzicka-Wolfenden, 1988) | 56.00628 |
| 319 | RADA880106 | Accessible surface area (Radzicka-Wolfenden, 1988) | 56.32041 |
| 320 | RADA880107 | Energy transfer from out to in(95%buried) (Radzicka-Wolfenden, 1988) | 60.98354 |
| 321 | RADA880108 | Mean polarity (Radzicka-Wolfenden, 1988) | 61.01062 |
| 322 | RICJ880101 | Relative preference value at N" (Richardson-Richardson, 1988) | 52.70256 |
| 323 | RICJ880102 | Relative preference value at N' (Richardson-Richardson, 1988) | 52.70256 |
| 324 | RICJ880103 | Relative preference value at N-cap (Richardson-Richardson, 1988) | 53.07626 |
| 325 | RICJ880104 | Relative preference value at N1 (Richardson-Richardson, 1988) | 54.72270 |
| 326 | RICJ880105 | Relative preference value at N2 (Richardson-Richardson, 1988) | 55.56759 |
| 327 | RICJ880106 | Relative preference value at N3 (Richardson-Richardson, 1988) | 51.47314 |
| 328 | RICJ880107 | Relative preference value at N4 (Richardson-Richardson, 1988) | 54.60897 |
| 329 | RICJ880108 | Relative preference value at N5 (Richardson-Richardson, 1988) | 64.46057 |
| 330 | RICJ880109 | Relative preference value at Mid (Richardson-Richardson, 1988) | 51.88475 |
| 331 | RICJ880110 | Relative preference value at C5 (Richardson-Richardson, 1988) | 55.11265 |
| 332 | RICJ880111 | Relative preference value at C4 (Richardson-Richardson, 1988) | 53.79658 |
| 333 | RICJ880112 | Relative preference value at C3 (Richardson-Richardson, 1988) | 60.95104 |
| 334 | RICJ880113 | Relative preference value at C2 (Richardson-Richardson, 1988) | 58.96880 |
| 335 | RICJ880114 | Relative preference value at C1 (Richardson-Richardson, 1988) | 55.02600 |
| 336 | RICJ880115 | Relative preference value at C-cap (Richardson-Richardson, 1988) | 58.44346 |
| 337 | RICJ880116 | Relative preference value at C' (Richardson-Richardson, 1988) | 55.70299 |
| 338 | RICJ880117 | Relative preference value at C" (Richardson-Richardson, 1988) | 52.85420 |
| 339 | ROBB760101 | Information measure for alpha-helix (Robson-Suzuki, 1976) | 51.89016 |
| 340 | ROBB760102 | Information measure for N-terminal helix (Robson-Suzuki, 1976) | 56.45581 |
| 341 | ROBB760103 | Information measure for middle helix (Robson-Suzuki, 1976) | 51.93891 |
| 342 | ROBB760104 | Information measure for C-terminal helix (Robson-Suzuki, 1976) | 58.50303 |
| 343 | ROBB760105 | Information measure for extended (Robson-Suzuki, 1976) | 55.67049 |
| 344 | ROBB760106 | Information measure for pleated-sheet (Robson-Suzuki, 1976) | 54.58731 |
| 345 | ROBB760107 | Information measure for extended without H-bond (Robson-Suzuki, 1976) | 57.23570 |
| 346 | ROBB760108 | Information measure for turn (Robson-Suzuki, 1976) | 52.88670 |
| 347 | ROBB760109 | Information measure for N-terminal turn (Robson-Suzuki, 1976) | 53.48245 |
| 348 | ROBB760110 | Information measure for middle turn (Robson-Suzuki, 1976) | 54.31109 |
| 349 | ROBB760111 | Information measure for C-terminal turn (Robson-Suzuki, 1976) | 53.31997 |
| 350 | ROBB760112 | Information measure for coil (Robson-Suzuki, 1976) | 53.16833 |
| 351 | ROBB760113 | Information measure for loop (Robson-Suzuki, 1976) | 53.11958 |
| 352 | ROBB790101 | Hydration free energy (Robson-Osguthorpe, 1979) | 55.34012 |
| 353 | ROSG850101 | Mean area buried on transfer (Rose et al., 1985) | 53.16833 |
| 354 | ROSG850102 | Mean fractional area loss (Rose et al., 1985) | 63.01993 |
| 355 | ROSM880101 | Side chain hydropathy, uncorrected for solvation (Roseman, 1988) | 55.71382 |
| 356 | ROSM880102 | Side chain hydropathy, corrected for solvation (Roseman, 1988) | 58.28098 |
| 357 | ROSM880103 | Loss of Side chain hydropathy by helix formation (Roseman, 1988) | 55.58384 |
| 358 | SIMZ760101 | Transfer free energy (Simon, 1976), Cited by Charton-Charton (1982) | 56.49372 |
| 359 | SNEP660101 | Principal component I (Sneath, 1966) | 54.86893 |
| 360 | SNEP660102 | Principal component II (Sneath, 1966) | 51.68436 |
| 361 | SNEP660103 | Principal component III (Sneath, 1966) | 56.72119 |
| 362 | SNEP660104 | Principal component IV (Sneath, 1966) | 58.94172 |
| 363 | SUEM840101 | Zimm-Bragg parameter s at 20 C (Sueki et al., 1984) | 52.32344 |
| 364 | SUEM840102 | Zimm-Bragg parameter sigma x 1.0E4 (Sueki et al., 1984) | 56.18501 |
| 365 | SWER830101 | Optimal matching hydrophobicity (Sweet-Eisenberg, 1983) | 53.93739 |
| 366 | TANS770101 | Normalized frequency of alpha-helix (Tanaka-Scheraga, 1977) | 53.22249 |
| 367 | TANS770102 | Normalized frequency of isolated helix (Tanaka-Scheraga, 1977) | 54.50065 |
| 368 | TANS770103 | Normalized frequency of extended structure (Tanaka-Scheraga, 1977) | 57.11114 |
| 369 | TANS770104 | Normalized frequency of chain reversal R (Tanaka-Scheraga, 1977) | 54.48440 |
| 370 | TANS770105 | Normalized frequency of chain reversal S (Tanaka-Scheraga, 1977) | 52.71880 |
| 371 | TANS770106 | Normalized frequency of chain reversal D (Tanaka-Scheraga, 1977) | 56.34207 |
| 372 | TANS770107 | Normalized frequency of left-handed helix (Tanaka-Scheraga, 1977) | 56.57496 |
| 373 | TANS770108 | Normalized frequency of zeta R (Tanaka-Scheraga, 1977) | 52.59965 |
| 374 | TANS770109 | Normalized frequency of coil (Tanaka-Scheraga, 1977) | 54.14861 |
| 375 | TANS770110 | Normalized frequency of chain reversal (Tanaka-Scheraga, 1977) | 54.86893 |
| 376 | VASM830101 | Relative population of conformational state A (Vasquez et al., 1983) | 52.32886 |
| 377 | VASM830102 | Relative population of conformational state C (Vasquez et al., 1983) | 54.92309 |
| 378 | VASM830103 | Relative population of conformational state E (Vasquez et al., 1983) | 54.09445 |
| 379 | VELV850101 | Electron-ion interaction potential (Veljkovic et al., 1985) | 54.65230 |
| 380 | VENT840101 | Bitterness (Venanzi, 1984) | 53.16833 |
| 381 | VHEG790101 | Transfer free energy to lipophilic phase (von Heijne-Blomberg, 1979) | 56.31499 |
| 382 | WARP780101 | Average interactions per side chain atom (Warme-Morgan, 1978) | 61.66594 |
| 383 | WEBA780101 | RF value in high salt chromatography (Weber-Lacey, 1978) | 58.56261 |
| 384 | WERD780101 | Propensity to be buried inside (Wertz-Scheraga, 1978) | 57.52816 |
| 385 | WERD780102 | Free energy change of epsilon(i) to epsilon(ex) (Wertz-Scheraga, 1978) | 54.50607 |
| 386 | WERD780103 | Free energy change of alpha(Ri) to alpha(Rh) (Wertz-Scheraga, 1978) | 55.30221 |
| 387 | WERD780104 | Free energy change of epsilon(i) to alpha(Rh) (Wertz-Scheraga, 1978) | 53.26581 |
| 388 | WOEC730101 | Polar requirement (Woese, 1973) | 57.39818 |
| 389 | WOLR810101 | Hydration potential (Wolfenden et al., 1981) | 55.69216 |
| 390 | WOLS870101 | Principal property value z1 (Wold et al., 1987) | 55.49718 |
| 391 | WOLS870102 | Principal property value z2 (Wold et al., 1987) | 56.62912 |
| 392 | WOLS870103 | Principal property value z3 (Wold et al., 1987) | 61.99090 |
| 393 | YUTK870101 | Unfolding Gibbs energy in water, pH7.0 (Yutani et al., 1987) | 55.89796 |
| 394 | YUTK870102 | Unfolding Gibbs energy in water, pH9.0 (Yutani et al., 1987) | 55.91421 |
| 395 | YUTK870103 | Activation Gibbs energy of unfolding, pH7.0 (Yutani et al., 1987) | 54.65771 |
| 396 | YUTK870104 | Activation Gibbs energy of unfolding, pH9.0 (Yutani et al., 1987) | 54.63063 |
| 397 | ZASB820101 | Dependence of partition coefficient on ionic strength (Zaslavsky et al., 1982) | 52.59424 |
| 398 | ZIMJ680101 | Hydrophobicity (Zimmerman et al., 1968) | 56.27708 |
| 399 | ZIMJ680102 | Bulkiness (Zimmerman et al., 1968) | 53.23332 |
| 400 | ZIMJ680103 | Polarity (Zimmerman et al., 1968) | 57.21404 |
| 401 | ZIMJ680104 | Isoelectric point (Zimmerman et al., 1968) | 62.94411 |
| 402 | ZIMJ680105 | RF rank (Zimmerman et al., 1968) | 56.69952 |
| 403 | AURR980101 | Normalized positional residue frequency at helix termini N4'(Aurora-Rose,1998) | 54.96101 |
| 404 | AURR980102 | Normalized positional residue frequency at helix termini N"' (Aurora-Rose,1998) | 56.41248 |
| 405 | AURR980103 | Normalized positional residue frequency at helix termini N" (Aurora-Rose,1998) | 53.03834 |
| 406 | AURR980104 | Normalized positional residue frequency at helix termini N'(Aurora-Rose,1998) | 57.20862 |
| 407 | AURR980105 | Normalized positional residue frequency at helix termini Nc (Aurora-Rose,1998) | 54.04571 |
| 408 | AURR980106 | Normalized positional residue frequency at helix termini N1 (Aurora-Rose,1998) | 52.9896 |
| 409 | AURR980107 | Normalized positional residue frequency at helix termini N2 (Aurora-Rose,1998) | 51.56521 |
| 410 | AURR980108 | Normalized positional residue frequency at helix termini N3 (Aurora-Rose,1998) | 54.17569 |
| 411 | AURR980109 | Normalized positional residue frequency at helix termini N4 (Aurora-Rose,1998) | 53.26040 |
| 412 | AURR980110 | Normalized positional residue frequency at helix termini N5 (Aurora-Rose,1998) | 53.31456 |
| 413 | AURR980111 | Normalized positional residue frequency at helix termini C5 (Aurora-Rose,1998) | 53.20082 |
| 414 | AURR980112 | Normalized positional residue frequency at helix termini C4 (Aurora-Rose,1998) | 52.71339 |
| 415 | AURR980113 | Normalized positional residue frequency at helix termini C3 (Aurora-Rose,1998) | 52.90836 |
| 416 | AURR980114 | Normalized positional residue frequency at helix termini C2 (Aurora-Rose,1998) | 58.10225 |
| 417 | AURR980115 | Normalized positional residue frequency at helix termini C1 (Aurora-Rose,1998) | 54.60897 |
| 418 | AURR980116 | Normalized positional residue frequency at helix termini Cc (Aurora-Rose,1998) | 55.93588 |
| 419 | AURR980117 | Normalized positional residue frequency at helix termini C' (Aurora-Rose,1998) | 58.57886 |
| 420 | AURR980118 | Normalized positional residue frequency at helix termini C" (Aurora-Rose,1998) | 57.40901 |
| 421 | AURR980119 | Normalized positional residue frequency at helix termini C"' (Aurora-Rose,1998) | 56.28250 |
| 422 | AURR980120 | Normalized positional residue frequency at helix termini C4' (Aurora-Rose,1998) | 53.86157 |
| 423 | ONEK900101 | Delta G values for the peptides extrapolated to 0 M urea (O'Neil-DeGrado,1990) | 55.67591 |
| 424 | ONEK900102 | Helix formation parameters (delta delta G) (O'Neil-DeGrado, 1990) | 56.58037 |
| 425 | VINM940101 | Normalized flexibility parameters (B-values), average (Vihinen et al., 1994) | 58.52470 |
| 426 | VINM940102 | Normalized flexibility parameters (B-values) for each residue surrounded by none rigid neighbours (Vihinen et al., 1994) | 54.93393 |
| 427 | VINM940103 | Normalized flexibility parameters (B-values) for each residue surrounded by one rigid neighbours (Vihinen et al., 1994) | 54.53856 |
| 428 | VINM940104 | Normalized flexibility parameters (B-values) for each residue surrounded by two rigid neighbours (Vihinen et al., 1994) | 61.07561 |
| 429 | MUNV940101 | Free energy in alpha-helical conformation (Munoz-Serrano, 1994) | 51.99848 |
| 430 | MUNV940102 | Free energy in alpha-helical region (Munoz-Serrano, 1994) | 52.69714 |
| 431 | MUNV940103 | Free energy in beta-strand conformation (Munoz-Serrano, 1994) | 54.62522 |
| 432 | MUNV940104 | Free energy in beta-strand region (Munoz-Serrano, 1994) | 54.18111 |
| 433 | MUNV940105 | Free energy in beta-strand region (Munoz-Serrano, 1994) | 53.44996 |
| 434 | WIMW960101 | Free energies of transfer of AcWl-X-LL peptides from bilayer interface to water (Wimley-White, 1996) | 54.17028 |
| 435 | KIMC930101 | Thermodynamic beta sheet propensity (Kim-Berg, 1993) | 53.12500 |
| 436 | MONM990101 | Turn propensity scale for transmembrane helices (Monne et al., 1999) | 55.69757 |
| 437 | BLAM930101 | Alpha helix propensity of position 44 in T4 lysozyme (Blaber et al., 1993) | 51.95516 |
| 438 | PARS000101 | p-Values of mesophilic proteins based on the distributions of B values (Parthasarathy-Murthy, 2000) | 55.70841 |
| 439 | PARS000102 | p-Values of thermophilic proteins based on the distributions of B values (Parthasarathy-Murthy, 2000) | 55.61633 |
| 440 | KUMS000101 | Distribution of amino acid residues in the 18 non-redundant families of thermophilic proteins (Kumar et al., 2000) | 54.22444 |
| 441 | KUMS000102 | Distribution of amino acid residues in the 18 non-redundant families of mesophilic proteins (Kumar et al., 2000) | 54.13237 |
| 442 | KUMS000103 | Distribution of amino acid residues in the alpha-helices in thermophilic proteins (Kumar et al., 2000) | 54.61438 |
| 443 | KUMS000104 | Distribution of amino acid residues in the alpha-helices in mesophilic proteins (Kumar et al., 2000) | 54.31109 |
| 444 | TAKK010101 | Side-chain contribution to protein stability (kJ/mol) (Takano-Yutani, 2001) | 54.48440 |
| 445 | FODM020101 | Propensity of amino acids within pi-helices (Fodje-Al-Karadaghi, 2002) | 54.48982 |
| 446 | NADH010101 | Hydropathy scale based on self-information values in the two-state model (5% accessibility) (Naderi-Manesh et al., 2001) | 55.69216 |
| 447 | NADH010102 | Hydropathy scale based on self-information values in the two-state model (9% accessibility) (Naderi-Manesh et al., 2001) | 63.08492 |
| 448 | NADH010103 | Hydropathy scale based on self-information values in the two-state model (16% accessibility) (Naderi-Manesh et al., 2001) | 60.84272 |
| 449 | NADH010104 | Hydropathy scale based on self-information values in the two-state model (20% accessibility) (Naderi-Manesh et al., 2001) | 59.32084 |
| 450 | NADH010105 | Hydropathy scale based on self-information values in the two-state model (25% accessibility) (Naderi-Manesh et al., 2001) | 56.97032 |
| 451 | NADH010106 | Hydropathy scale based on self-information values in the two-state model (36% accessibility) (Naderi-Manesh et al., 2001) | 55.74632 |
| 452 | NADH010107 | Hydropathy scale based on self-information values in the two-state model (50% accessibility) (Naderi-Manesh et al., 2001) | 54.05113 |
| 453 | MONM990201 | Averaged turn propensities in a transmembrane helix (Monne et al., 1999) | 53.24415 |
| 454 | KOEP990101 | Alpha-helix propensity derived from designed sequences (Koehl-Levitt, 1999) | 53.53661 |
| 455 | KOEP990102 | Beta-sheet propensity derived from designed sequences (Koehl-Levitt, 1999) | 56.59662 |
| 456 | CEDJ970101 | Composition of amino acids in extracellular proteins (percent) (Cedano et al., 1997) | 54.35984 |
| 457 | CEDJ970102 | Composition of amino acids in anchored proteins (percent) (Cedano et al., 1997) | 53.90490 |
| 458 | CEDJ970103 | Composition of amino acids in membrane proteins (percent) (Cedano et al., 1997) | 54.13237 |
| 459 | CEDJ970104 | Composition of amino acids in intracellular proteins (percent) (Cedano et al., 1997) | 54.15945 |
| 460 | CEDJ970105 | Composition of amino acids in nuclear proteins (percent) (Cedano et al., 1997) | 55.52968 |
| 461 | FUKS010101 | Surface composition of amino acids in intracellular proteins of thermophiles (percent) (Fukuchi-Nishikawa, 2001) | 60.08990 |
| 462 | FUKS010102 | Surface composition of amino acids in intracellular proteins of mesophiles (percent) (Fukuchi-Nishikawa, 2001) | 58.12933 |
| 463 | FUKS010103 | Surface composition of amino acids in extracellular proteins of mesophiles (percent) (Fukuchi-Nishikawa, 2001) | 58.09142 |
| 464 | FUKS010104 | Surface composition of amino acids in nuclear proteins (percent) (Fukuchi-Nishikawa, 2001) | 63.23657 |
| 465 | FUKS010105 | Interior composition of amino acids in intracellular proteins of thermophiles (percent) (Fukuchi-Nishikawa, 2001) | 54.16486 |
| 466 | FUKS010106 | Interior composition of amino acids in intracellular proteins of mesophiles (percent) (Fukuchi-Nishikawa, 2001) | 53.65576 |
| 467 | FUKS010107 | Interior composition of amino acids in extracellular proteins of mesophiles (percent) (Fukuchi-Nishikawa, 2001) | 56.23375 |
| 468 | FUKS010108 | Interior composition of amino acids in nuclear proteins (percent) (Fukuchi-Nishikawa, 2001) | 55.88713 |
| 469 | FUKS010109 | Entire chain composition of amino acids in intracellular proteins of thermophiles (percent) (Fukuchi-Nishikawa, 2001) | 55.96295 |
| 470 | FUKS010110 | Entire chain composition of amino acids in intracellular proteins of mesophiles (percent) (Fukuchi-Nishikawa, 2001) | 54.68479 |
| 471 | FUKS010111 | Entire chain composition of amino acids in extracellular proteins of mesophiles (percent) (Fukuchi-Nishikawa, 2001) | 53.23332 |
| 472 | FUKS010112 | Entire chain compositino of amino acids in nuclear proteins (percent) (Fukuchi-Nishikawa, 2001) | 54.85810 |
| 473 | AVBF000101 | Screening coefficients gamma, local (Avbelj, 2000) | 53.94281 |
| 474 | AVBF000102 | Screening coefficients gamma, non-local (Avbelj, 2000) | 52.98419 |
| 475 | AVBF000103 | Slopes tripeptide, FDPB VFF neutral (Avbelj, 2000) | 54.41941 |
| 476 | AVBF000104 | Slopes tripeptides, LD VFF neutral (Avbelj, 2000) | 52.71880 |
| 477 | AVBF000105 | Slopes tripeptide, FDPB VFF noside (Avbelj, 2000) | 55.32929 |
| 478 | AVBF000106 | Slopes tripeptide FDPB VFF all (Avbelj, 2000) | 57.31694 |
| 479 | AVBF000107 | Slopes tripeptide FDPB PARSE neutral (Avbelj, 2000) | 54.53856 |
| 480 | AVBF000108 | Slopes dekapeptide, FDPB VFF neutral (Avbelj, 2000) | 55.63258 |
| 481 | AVBF000109 | Slopes proteins, FDPB VFF neutral (Avbelj, 2000) | 56.55329 |
| 482 | YANJ020101 | Side-chain conformation by gaussian evolutionary method (Yang et al., 2002) | 52.55091 |
| 483 | MITS020101 | Amphiphilicity index (Mitaku et al., 2002) | 60.36070 |
| 484 | TSAJ990101 | Volumes including the crystallographic waters using the ProtOr (Tsai et al., 1999) | 54.80394 |
| 485 | TSAJ990102 | Volumes not including the crystallographic waters using the ProtOr (Tsai et al., 1999) | 54.91768 |
| 486 | COSI940101 | Electron-ion interaction potential values (Cosic, 1994) | 54.66313 |
| 487 | PONP930101 | Hydrophobicity scales (Ponnuswamy, 1993) | 56.23917 |
| 488 | WILM950101 | Hydrophobicity coefficient in RP-HPLC, C18 with 0.1%TFA/MeCN/H2O (Wilce et al. 1995) | 59.90576 |
| 489 | WILM950102 | Hydrophobicity coefficient in RP-HPLC, C8 with 0.1%TFA/MeCN/H2O (Wilce et al. 1995) | 57.93436 |
| 490 | WILM950103 | Hydrophobicity coefficient in RP-HPLC, C4 with 0.1%TFA/MeCN/H2O (Wilce et al. 1995) | 61.42223 |
| 491 | WILM950104 | Hydrophobicity coefficient in RP-HPLC, C18 with 0.1%TFA/2-PrOH/MeCN/H2O (Wilce et al. 1995) | 52.58341 |
| 492 | KUHL950101 | Hydrophilicity scale (Kuhn et al., 1995) | 55.39970 |
| 493 | GUOD860101 | Retention coefficient at pH 2 (Guo et al., 1986) | 60.43111 |
| 494 | JURD980101 | Modified Kyte-Doolittle hydrophobicity scale (Juretic et al., 1998) | 57.57691 |
| 495 | BASU050101 | Interactivity scale obtained from the contact matrix (Bastolla et al., 2005) | 54.27318 |
| 496 | BASU050102 | Interactivity scale obtained by maximizing the mean of correlation coefficient over single-domain globular proteins (Bastolla et al., 2005) | 54.30026 |
| 497 | BASU050103 | Interactivity scale obtained by maximizing the mean of correlation coefficient over pairs of sequences sharing the TIM barrel fold (Bastolla et al., 2005) | 55.78964 |
| 498 | SUYM030101 | Linker propensity index (Suyama-Ohara, 2003) | 52.84337 |
| 499 | PUNT030101 | Knowledge-based membrane-propensity scale from 1D_Helix in MPtopo databases (Punta-Maritan, 2003) | 58.20516 |
| 500 | PUNT030102 | Knowledge-based membrane-propensity scale from 3D_Helix in MPtopo databases (Punta-Maritan, 2003) | 55.95212 |
| 501 | GEOR030101 | Linker propensity from all dataset (George-Heringa, 2003) | 54.44649 |
| 502 | GEOR030102 | Linker propensity from 1-linker dataset (George-Heringa, 2003) | 55.12348 |
| 503 | GEOR030103 | Linker propensity from 2-linker dataset (George-Heringa, 2003) | 54.24068 |
| 504 | GEOR030104 | Linker propensity from 3-linker dataset (George-Heringa, 2003) | 54.27318 |
| 505 | GEOR030105 | Linker propensity from small dataset (linker length is less than six residues) (George-Heringa, 2003) | 64.54376 |
| 506 | GEOR030106 | Linker propensity from medium dataset (linker length is between six and 14 residues) (George-Heringa, 2003) | 53.35789 |
| 507 | GEOR030107 | Linker propensity from long dataset (linker length is greater than 14 residues) (George-Heringa, 2003) | 54.76603 |
| 508 | GEOR030108 | Linker propensity from helical (annotated by DSSP) dataset (George-Heringa, 2003) | 53.92656 |
| 509 | GEOR030109 | Linker propensity from non-helical (annotated by DSSP) dataset (George-Heringa, 2003) | 53.54744 |
| 510 | ZHOH040101 | The stability scale from the knowledge-based atom-atom potential (Zhou-Zhou, 2004) | 53.95906 |
| 511 | ZHOH040102 | The relative stability scale extracted from mutation experiments (Zhou-Zhou, 2004) | 53.60702 |
| 512 | ZHOH040103 | Buriability (Zhou-Zhou, 2004) | 58.25932 |
| 513 | BAEK050101 | Linker index (Bae et al., 2005) | 55.65425 |
| 514 | HARY940101 | Mean volumes of residues buried in protein interiors (Harpaz et al., 1994) | 54.90685 |
| 515 | PONJ960101 | Average volumes of residues (Pontius et al., 1996) | 53.67743 |
| 516 | DIGM050101 | Hydrostatic pressure asymmetry index, PAI (Di Giulio, 2005) | 54.68479 |
| 517 | WOLR790101 | Hydrophobicity index (Wolfenden et al., 1979) | 55.76256 |
| 518 | OLSK800101 | Average internal preferences (Olsen, 1980) | 59.0338 |
| 519 | KIDA850101 | Hydrophobicity-related index (Kidera et al., 1985) | 60.17114 |
| 520 | GUYH850102 | Apparent partition energies calculated from Wertz-Scheraga index (Guy, 1985) | 57.38735 |
| 521 | GUYH850103 | Apparent partition energies calculated from Robson-Osguthorpe index (Guy, 1985) | 55.3672 |
| 522 | GUYH850104 | Apparent partition energies calculated from Janin index (Guy, 1985) | 64.18284 |
| 523 | GUYH850105 | Apparent partition energies calculated from Chothia index (Guy, 1985) | 59.33167 |
| 524 | ROSM880104 | Hydropathies of amino acid side chains, neutral form (Roseman, 1988) | 56.37998 |
| 525 | ROSM880105 | Hydropathies of amino acid side chains, pi-values in pH 7.0 (Roseman, 1988) | 56.94866 |
| 526 | JACR890101 | Weights from the IFH scale (Jacobs-White, 1989) | 58.51928 |
| 527 | COWR900101 | Hydrophobicity index, 3.0 pH (Cowan-Whittaker, 1990) | 60.73982 |
| 528 | BLAS910101 | Scaled side chain hydrophobicity values (Black-Mould, 1991) | 55.32929 |
| 529 | CASG920101 | Hydrophobicity scale from native protein structures (Casari-Sippl, 1992) | 57.46317 |
| 530 | CORJ870101 | NNEIG index (Cornette et al., 1987) | 56.76993 |
| 531 | CORJ870102 | SWEIG index (Cornette et al., 1987) | 53.96989 |
| 532 | CORJ870103 | PRIFT index (Cornette et al., 1987) | 56.24458 |
| 533 | CORJ870104 | PRILS index (Cornette et al., 1987) | 56.06044 |
| 534 | CORJ870105 | ALTFT index (Cornette et al., 1987) | 58.87673 |
| 535 | CORJ870106 | ALTLS index (Cornette et al., 1987) | 58.46512 |
| 536 | CORJ870107 | TOTFT index (Cornette et al., 1987) | 57.84770 |
| 537 | CORJ870108 | TOTLS index (Cornette et al., 1987) | 57.33860 |
| 538 | MIYS990101 | Relative partition energies derived by the Bethe approximation (Miyazawa-Jernigan, 1999) | 57.53358 |
| 539 | MIYS990102 | Optimized relative partition energies - method A (Miyazawa-Jernigan, 1999) | 57.88020 |
| 540 | MIYS990103 | Optimized relative partition energies - method B (Miyazawa-Jernigan, 1999) | 59.98159 |
| 541 | MIYS990104 | Optimized relative partition energies - method C (Miyazawa-Jernigan, 1999) | 60.23614 |
| 542 | MIYS990105 | Optimized relative partition energies - method D (Miyazawa-Jernigan, 1999) | 60.06282 |
| 543 | ENGD860101 | Hydrophobicity index (Engelman et al., 1986) | 58.34055 |
| 544 | FASG890101 | Hydrophobicity index (Fasman, 1989) | 61.21101 |
