# Supplementary material for: Position-Specific Analysis and Prediction for Protein Lysine Acetylation Based on Multiple Features
Source: PLoS One. 2012 Nov 16;7(11):e49108. doi: 10.1371/journal.pone.0049108 (PMC3500252; doi:10.1371/journal.pone.0049108)
Supplement: Table S3 — The predictive performance of the models trained with various features with an IG window size of 9. (DOC) [file pone.0049108.s003.doc]

**Table S3.** The predictive performance of the models trained with various features with an IG window size of 9.

| Training features | The performance of the prediction (%) | | | |
| --- | --- | --- | --- | --- |
|  | Accuracy | Sensitivity | Specificity | MCC |
| BE | 69.95±0.07 | 59.16±0.24 | 80.74±0.34 | 40.87±0.18 |
| KNN | 75.12±0.21 | 72.26±1.28 | 77.99±1.30 | 50.35±0.43 |
| AASA | 64.94±0.14 | 63.32±0.33 | 66.57±0.25 | 29.91±0.27 |
| BE+KNN+AASA | 76.69±0.14 | 75.00±0.35 | 78.38±0.35 | 53.42±0.29 |
