# Supplementary material for: Position-Specific Analysis and Prediction for Protein Lysine Acetylation Based on Multiple Features
Source: PLoS One. 2012 Nov 16;7(11):e49108. doi: 10.1371/journal.pone.0049108 (PMC3500252; doi:10.1371/journal.pone.0049108)
Supplement: Table S4 — The predictive performance of the models trained with various features with an IG window size of 11. (DOC) [file pone.0049108.s004.doc]

**Table S4.** The predictive performance of the models trained with various features with an IG window size of 11.

| Training features | The performance of the prediction (%) | | | |
| --- | --- | --- | --- | --- |
|  | Accuracy | Sensitivity | Specificity | MCC |
| BE | 69.97±0.08 | 61.57±0.53 | 78.37±0.56 | 40.51±0.18 |
| KNN | 74.97±0.17 | 72.59±0.31 | 77.35±0.34 | 50.00±0.34 |
| AASA | 65.11±0.14 | 63.30±0.40 | 66.92±0.52 | 30.24±0.28 |
| BE+KNN+AASA | 77.56±0.23 | 75.60±0.14 | 79.51±0.46 | 55.15±0.47 |
