# Supplementary material for: Position-Specific Analysis and Prediction for Protein Lysine Acetylation Based on Multiple Features
Source: PLoS One. 2012 Nov 16;7(11):e49108. doi: 10.1371/journal.pone.0049108 (PMC3500252; doi:10.1371/journal.pone.0049108)
Supplement: Table S5 — The predictive performance of the models trained with various features with an IG window size of 15. (DOC) [file pone.0049108.s005.doc]

**Table S5.** The predictive performance of the models trained with various features with an IG window size of 15.

| Training features | The performance of the prediction (%) | | | |
| --- | --- | --- | --- | --- |
|  | Accuracy | Sensitivity | Specificity | MCC |
| BE | 68.85±0.16 | 64.10±0.17 | 73.61±0.27 | 37.88±0.32 |
| KNN | 74.85±0.10 | 72.84±0.34 | 76.87±0.25 | 49.75±0.19 |
| AASA | 65.39±0.16 | 62.77±0.51 | 68.01±0.47 | 30.82±0.31 |
| BE+KNN+AASA | 76.97±0.10 | 75.47±0.19 | 78.46±0.12 | 53.96±0.19 |
