# Supplementary material for: Position-Specific Analysis and Prediction for Protein Lysine Acetylation Based on Multiple Features
Source: PLoS One. 2012 Nov 16;7(11):e49108. doi: 10.1371/journal.pone.0049108 (PMC3500252; doi:10.1371/journal.pone.0049108)
Supplement: Table S6 — The predictive performance of the models trained with various features with an IG window size of 17. (DOC) [file pone.0049108.s006.doc]

**Table S6.** The predictive performance of the models trained with various features with an IG window size of 17.

| Training features | The performance of the prediction (%) | | | |
| --- | --- | --- | --- | --- |
|  | Accuracy | Sensitivity | Specificity | MCC |
| BE | 69.40±0.18 | 64.13±0.13 | 74.67±0.35 | 39.01±0.37 |
| KNN | 73.72±0.25 | 58.87±0.43 | 88.56±0.25 | 49.67±0.49 |
| AASA | 65.40±0.20 | 63.16±0.34 | 67.65±0.52 | 30.84±0.40 |
| BE+KNN+AASA | 76.76±0.21 | 74.17±0.24 | 79.36±0.35 | 53.60±0.42 |
