# Supplementary material for: Position-Specific Analysis and Prediction for Protein Lysine Acetylation Based on Multiple Features
Source: PLoS One. 2012 Nov 16;7(11):e49108. doi: 10.1371/journal.pone.0049108 (PMC3500252; doi:10.1371/journal.pone.0049108)
Supplement: Table S7 — The MCC of model trained with different features is compared via P -values on the paired Welch’s t-test. For the entry at row i, column j of the table, there is statistical difference when P≤0.05, or else there isn’t significantly different. (DOC) [file pone.0049108.s007.doc]

**Table S7. The MCC of model trained with different features is compared via *P*-values on the paired Welch’s t-test.** For the entry at row *i*, column *j* of the table, there is statistical difference when *P*$\leq$0.05, or else there isn’t significantly different.

|  | BE | KNN | AASA | BE+KNN+AASA |
| --- | --- | --- | --- | --- |
| BE | 1.00 | 7.83e-10 | 4.79e-09 | 1.33e-13 |
| KNN |  | 1.00 | 4.30e-11 | 1.47e-08 |
| AASA |  |  | 1.00 | 1.12e-14 |
| BE+KNN+AASA |  |  |  | 1.00 |
