# Supplementary material for: Position-Specific Analysis and Prediction for Protein Lysine Acetylation Based on Multiple Features
Source: PLoS One. 2012 Nov 16;7(11):e49108. doi: 10.1371/journal.pone.0049108 (PMC3500252; doi:10.1371/journal.pone.0049108)
Supplement: Table S8 — The MCC of models with different IG window size is compared via P -values on the paired Welch’s t-test. (DOC) [file pone.0049108.s008.doc]

**Table S8. The MCC of models with different IG window size is compared via *P*-values on the paired Welch’s t-test.**

|  | 9 | 11 | 13 | 15 | 17 |
| --- | --- | --- | --- | --- | --- |
| 9 | 1.00 | 2.36e-04 | 2.98e-09 | 1.38e-02 | 0.49 |
| 11 |  | 1.00 | 3.01e-07 | 1.58e-03 | 1.22e-03 |
| 13 |  |  | 1.00 | 2.32e-09 | 1.79e-08 |
| 15 |  |  |  | 1.00 | 0.17 |
| 17 |  |  |  |  | 1.00 |
