# Supplementary material for: Position-Specific Analysis and Prediction for Protein Lysine Acetylation Based on Multiple Features
Source: PLoS One. 2012 Nov 16;7(11):e49108. doi: 10.1371/journal.pone.0049108 (PMC3500252; doi:10.1371/journal.pone.0049108)
Supplement: Table S9 — The MCC of models with different general window size is compared via P -values on the paired Welch’s t-test. (DOC) [file pone.0049108.s009.doc]

**Table S9. The MCC of models with different general window size is compared via *P*-values on the paired Welch’s t-test.**

|  | 9 | 11 | 13 | 15 | 17 | 19 | 21 |
| --- | --- | --- | --- | --- | --- | --- | --- |
| 9 | 1.00 | 5.50e-06 | 2.72e-06 | 5.17e-08 | 7.09e-08 | 1.99e-08 | 2.81e-08 |
| 11 |  | 1.00 | 9.73e-03 | 6.01e-06 | 9.69e-06 | 1.58e-06 | 4.78e-06 |
| 13 |  |  | 1.00 | 1.68e-03 | 2.46e-03 | 9.94e-04 | 1.06e-02 |
| 15 |  |  |  | 1.00 | 0.83 | 0.92 | 1.70e-02 |
| 17 |  |  |  |  | 1.00 | 0.87 | 3.59e-02 |
| 19 |  |  |  |  |  | 1.00 | 3.05e-03 |
| 21 |  |  |  |  |  |  | 1.00 |
