# Supplementary material for: Position-Specific Analysis and Prediction for Protein Lysine Acetylation Based on Multiple Features
Source: PLoS One. 2012 Nov 16;7(11):e49108. doi: 10.1371/journal.pone.0049108 (PMC3500252; doi:10.1371/journal.pone.0049108)
Supplement: Table S10 — The MCC of our method and other prediction methods is compared via P -values on the paired Welch’s t-test. (DOC) [file pone.0049108.s010.doc]

**Table S10. The MCC of our method and other prediction methods is compared via *P*-values on the paired Welch’s t-test.**

|  | LysAcet | EnsemblePail | Phosida | PSKAcePred |
| --- | --- | --- | --- | --- |
| LysAcet | 1.00 | 1.25e-03 | 2.12e-08 | 4.69e-11 |
| EnsemblePail |  | 1.00 | 1.85e-07 | 1.11e-10 |
| Phosida |  |  | 1.00 | 5.46e-10 |
| PSKAcePred |  |  |  | 1.00 |
